# Development and Use of Mobile Messaging for Individuals With Musculoskeletal Pain Conditions: Scoping Review

**DOI:** 10.2196/55625

**Published:** 2024-08-14

**Authors:** Nigel Armfield, Rachel Elphinston, Jenna Liimatainen, Simone Scotti Requena, Chloe-Emily Eather, Sisira Edirippulige, Carrie Ritchie, Sarah Robins, Michele Sterling

**Affiliations:** 1 RECOVER Injury Research Centre The University of Queensland Brisbane Australia; 2 National Health and Medical Research Council (NHMRC) Centre for Research Excellence in Better Outcomes for Compensable Injury Brisbane Australia; 3 Centre for Health Services Research Faculty of Medicine The University of Queensland Brisbane Australia; 4 STARS Education and Research Alliance Surgical Treatment and Rehabilitation Service The University of Queensland and Metro North Health Brisbane Australia; 5 School of Psychology The University of Queensland St Lucia Australia; 6 Centre for Mental Health, Melbourne School of Population and Global Health, The University of Melbourne Melbourne Australia; 7 School of Health and Rehabilitation Sciences The University of Queensland St Lucia Australia; 8 Centre for Online Health, Centre for Health Services Research, Faculty of Medicine, The University of Queensland Brisbane Australia

**Keywords:** musculoskeletal, pain, SMS text messaging, mobile health, mHealth, intervention design, design, scoping review, musculoskeletal pain, development, mobile messaging, behavior change, efficacy, effectiveness, messaging, implementation, sustainability, mobile phone

## Abstract

**Background:**

Population studies show that musculoskeletal conditions are a leading contributor to the total burden of healthy life lost, second only to cancer and with a similar burden to cardiovascular disease. Prioritizing the delivery of effective treatments is necessary, and with the ubiquity of consumer smart devices, the use of digital health interventions is increasing. Messaging is popular and easy to use and has been studied for a range of health-related uses, including health promotion, encouragement of behavior change, and monitoring of disease progression. It may have a useful role to play in the management and self-management of musculoskeletal conditions.

**Objective:**

Previous reviews on the use of messaging for people with musculoskeletal conditions have focused on synthesizing evidence of effectiveness from randomized controlled trials. In this review, our objective was to map the musculoskeletal messaging literature more broadly to identify information that may inform the design of future messaging interventions and summarize the current evidence of efficacy, effectiveness, and economics.

**Methods:**

Following a prepublished protocol developed using the *Joanna Briggs Institute Manual for Evidence Synthesis*, we conducted a comprehensive scoping review of the literature (2010-2022; sources: PubMed, CINAHL, Embase, and PsycINFO) related to SMS text messaging and app-based messaging for people with musculoskeletal conditions. We described our findings using tables, plots, and a narrative summary.

**Results:**

We identified a total of 8328 papers for screening, of which 50 (0.6%) were included in this review (3/50, 6% previous reviews and 47/50, 94% papers describing 40 primary studies). Rheumatic diseases accounted for the largest proportion of the included primary studies (19/40, 48%), followed by studies on multiple musculoskeletal conditions or pain sites (10/40, 25%), back pain (9/40, 23%), neck pain (1/40, 3%), and “other” (1/40, 3%). Most studies (33/40, 83%) described interventions intended to promote positive behavior change, typically by encouraging increased physical activity and exercise. The studies evaluated a range of outcomes, including pain, function, quality of life, and medication adherence. Overall, the results either favored messaging interventions or had equivocal outcomes. While the theoretical underpinnings of the interventions were generally well described, only 4% (2/47) of the papers provided comprehensive descriptions of the messaging intervention design and development process. We found no relevant economic evaluations.

**Conclusions:**

Messaging has been used for the care and self-management of a range of musculoskeletal conditions with generally favorable outcomes reported. However, with few exceptions, design considerations are poorly described in the literature. Further work is needed to understand and disseminate information about messaging content and message delivery characteristics, such as timing and frequency specifically for people with musculoskeletal conditions. Similarly, further work is needed to understand the economic effects of messaging and practical considerations related to implementation and sustainability.

**International Registered Report Identifier (IRRID):**

RR2-10.1136/bmjopen-2021-048964

## Introduction

### Background

Musculoskeletal conditions, those affecting the bones, muscles, and joints, are recognized as a global public health problem, although the prevalence and burden of healthy life lost are difficult to estimate with certainty because population studies are few [1]. Where representative studies have been conducted, they have consistently shown high prevalence of musculoskeletal conditions that increases with age and has a greater burden on female than male individuals [2-5]. In the *Health Survey for England* 2018, a total of 17% of adults reported having a long-term musculoskeletal condition (19.5% female vs 14.2% male), with prevalence increasing with age (4.7% at the ages of 16-24 years vs 39% at the age of ≥85 years). A total of 80% of people who reported having a long-term musculoskeletal condition also reported chronic pain (pain for >3 months), with 34.8% reporting pain that highly interfered with their life activities [4,5]. The Australian *National Health Survey* 2017 to 2018 reported that 29% of Australians were living with a chronic musculoskeletal condition (age standardized; adults aged ≥45 years: 51%; 55.3% female vs 47.3% male) [3]. musculoskeletal conditions were the second leading contributor to total burden of healthy life lost, equal to the burden of cardiovascular disease (13% of total burden in disability-adjusted life years), second only to cancer (18% of total burden) [2]. Prioritizing the delivery of effective treatments is necessary to address the substantial burden of musculoskeletal conditions.

With the ubiquity of consumer devices such as smartphones and tablets, technology may have a useful role to play in the management and self-management of musculoskeletal conditions; potentially improve accessibility of health care; and, in some circumstances, ease health system pressures. The use of technology for providing health-related activities is typically described as “digital health” and, more specifically, “mobile health” (mHealth) when referring to the use of mobile devices. While still a relatively new field, mHealth already has a considerable literature base, with examples of its use across most health disciplines and across the continuum of care from health promotion and prevention [6,7] to screening and diagnosis [8,9], therapy [10,11], and self-management [12,13] to cancer survivorship and palliative care [14,15]. While mHealth shows promise in improving aspects of health care, evidence to date is mixed, and caution is needed in interpreting the clinical value of mHealth for patients [16].

In this review, we focused on the development and use of mHealth for individuals with musculoskeletal pain conditions and specifically on health-related interactions that use text messaging as the delivery mechanism (SMS text messaging or messages provided via app-based push notifications), either alone or alongside another intervention. As one of the mobile technologies that have been established for longer, text messaging is familiar, easy to use, convenient, low cost, and available to anyone with a mobile device [17]. Messaging can be used as a vehicle to promote behavior change and guide self-management through prompts, reinforcement, reminders, activity recording, feedback, and adaptivity to the individual [17,18]. The effectiveness of messaging interventions has been assessed for a wide range of health problems, such as medication adherence and lifestyle change in diabetes; encouraging abstinence in smoking cessation; and, more recently, to encourage prevention behaviors during the COVID-19 pandemic [17,19-21].

In total, 2 previous reviews have explored the effectiveness of text messaging–based interventions for musculoskeletal conditions [22,23]. In a broad review of 19 randomized controlled trials (RCTs; 1086 participants) [23], 5 studies involved aspects of messaging [24-28], with 4 studies reporting improvements in pain [25-28] and functional disability [24-27] favoring digital interventions but not specifically favoring the messaging components [23]. A second review focused specifically on the effectiveness of text messaging–delivered interventions included 11 RCTs (1607 participants) [22]. Of the included studies, 5 assessed text messaging as an adjunct to usual care on treatment adherence and found improvements favoring text messaging [29-33]. In a further 5 RCTs, the effectiveness of text messaging as 1 component of a complex intervention was assessed [34-38], finding small but inconsistent effects on pain, functioning, adherence, and quality of life. In 1 RCT, text messaging was compared to telephone counseling, and similar effects on functioning were reported [39].

### Objectives

These previous reviews focused on intervention effectiveness and synthesized data from RCTs only. The findings of observational studies have not been synthesized, and these studies may contain useful information to inform and, ultimately, improve the effectiveness and adoption of future musculoskeletal interventions delivered using text messaging. Furthermore, important characteristics of interventions, such as the configuration of digital content, method of presentation, dose, frequency, and preferences, have not been synthesized. Consequently, to inform the design, development, and evaluation of future messaging interventions for people with musculoskeletal pain, we need to explore the literature using a wider lens. Therefore, in this study focused on individuals with musculoskeletal pain conditions, we had three aims: (1) to map the literature related to the use of mobile messaging; (2) to identify information that could be useful in the design of future messaging interventions; and (3) to explore and summarize the findings on efficacy, effectiveness, and economics derived from previous experimental and observational messaging studies.

## Methods

We designed and conducted this review according to a preregistered and published protocol [40] developed using the *Joanna Briggs Institute Manual for Evidence Synthesis* [41] and the PRISMA-ScR (Preferred Reporting Items for Systematic Reviews and Meta-Analyses extension for Scoping Reviews) [42] guidelines. The methods are described in full in the published protocol and summarized in brief in the following sections.

### Review Questions

Research question (RQ) 1 was as follows: In the context of musculoskeletal pain conditions, for which individuals, with which problems, and for what purpose, has messaging on mobile devices been used (eg, medication reminders, alerts, education, motivation, prevention, and data collection)?

RQ 2 was as follows: What information exists to guide the development of mobile messaging for musculoskeletal pain conditions (eg, frequency of messages, length of messages, duration of the intervention, and theoretical basis)?

RQ 3 was as follows: How have patients’ preferences been included in the design of a study, and how have their preferences been assessed?

RQ 4 was as follows: What methods have been used to evaluate the use of mobile messaging for musculoskeletal pain conditions (eg, how were outcomes assessed and what processes were involved)?

RQ 5 was as follows: Does the literature support the efficacy, effectiveness, and economics of messaging on mobile devices for individuals with musculoskeletal pain conditions?

### Inclusion Criteria

#### Participants

We included studies on adult participants with acute or chronic musculoskeletal pain conditions.

#### Concept

The concepts of interest were the development or evaluation of patient-focused health-related messaging (eg, SMS text messaging and app push notifications) provided on mobile devices such as smartphones and tablets.

#### Context

We included articles that described messaging used in any setting either as a primary intervention or as an adjunct to other interventions. We excluded studies focused on spinal cord injury, traumatic brain injury, moderate to severe orthopedic injuries, surgical patients, and conditions related to mobile phone overuse. We also excluded studies focused on health conditions primarily unrelated to the bones, muscles, and connective tissue (eg, diabetes, asthma, cancer, and stroke).

### Data Sources

We searched PubMed, CINAHL (via EBSCOhost), Embase, and PsycINFO (via APA PsycNET) using a strategy that combined controlled-vocabulary and free-text search terms related to messaging and musculoskeletal concepts. Because of resource limitations, we were unable to include gray literature in our searches.

### Search Strategy

The search strategy is described in detail in the published protocol [40], and the search queries are provided again in this paper in Multimedia Appendix 1. The search strategy was developed through discussion among the team and an iterative process of pilot searches. The final searches were conducted by SSR. Because of resource limitations, we restricted our searches to articles published in English, and because the area of digital health is a rapidly changing field, we limited our searches to articles published in the previous 10 years.

### Study Selection

We exported search results to EndNote (version X9; Clarivate Analytics) and Covidence (Veritas Health Innovation) [43] for duplicate removal and to manage the screening, selection, and record-keeping processes. We conducted study selection in 3 phases. First, using the predefined inclusion and exclusion criteria, 2 independent reviewers (from a pool of 7; SSR, JL, CEE, RE, CR, SR, and NA) screened the titles and abstracts to identify candidate articles for inclusion and to discard irrelevant articles. Second, 2 reviewers from the same pool reviewed the full text of each candidate article. Third, we searched the reference lists of the included papers to identify any further articles. At all stages, conflicts were resolved using a third reviewer from our pool.

### Data Extraction

Data were extracted by one reviewer (JL) and independently confirmed by 2 others (NA and CEE). Data were extracted using predefined extraction forms, as described in the protocol [40].

### Synthesis and Reporting

We described the results of the study selection process using a PRISMA (Preferred Reporting Items for Systematic Reviews and Meta-Analyses) flow diagram [44], with findings reported in accordance with the PRISMA-ScR checklist [42]. For each of our 5 review questions, we structured our findings using tables adapted from the Joanna Briggs Institute manual [41] refined as necessary at synthesis stage [40]. We then developed a narrative summary of the evidence for each of our review questions.

### Protocol Deviations

There were 4 minor protocol deviations. First, we excluded studies that described the use of mobile messaging to collect data in cases in which those data were not subsequently used to inform care or self-management (eg, studies that simply tested the feasibility of using text messaging to collect data and studies that used text messaging as a data collection method to model recovery trajectories). Second, we included study protocols associated with evaluation studies if they provided useful information about messaging design and development. Third, we classified the level of development of the country in which the study was conducted using the Human Development Index (HDI) [45]. Finally, we reran our searches in 2022 and, therefore, included studies from a 12-year period rather than the originally specified 10 years.

## Results

### Overview

**Figure 1:**
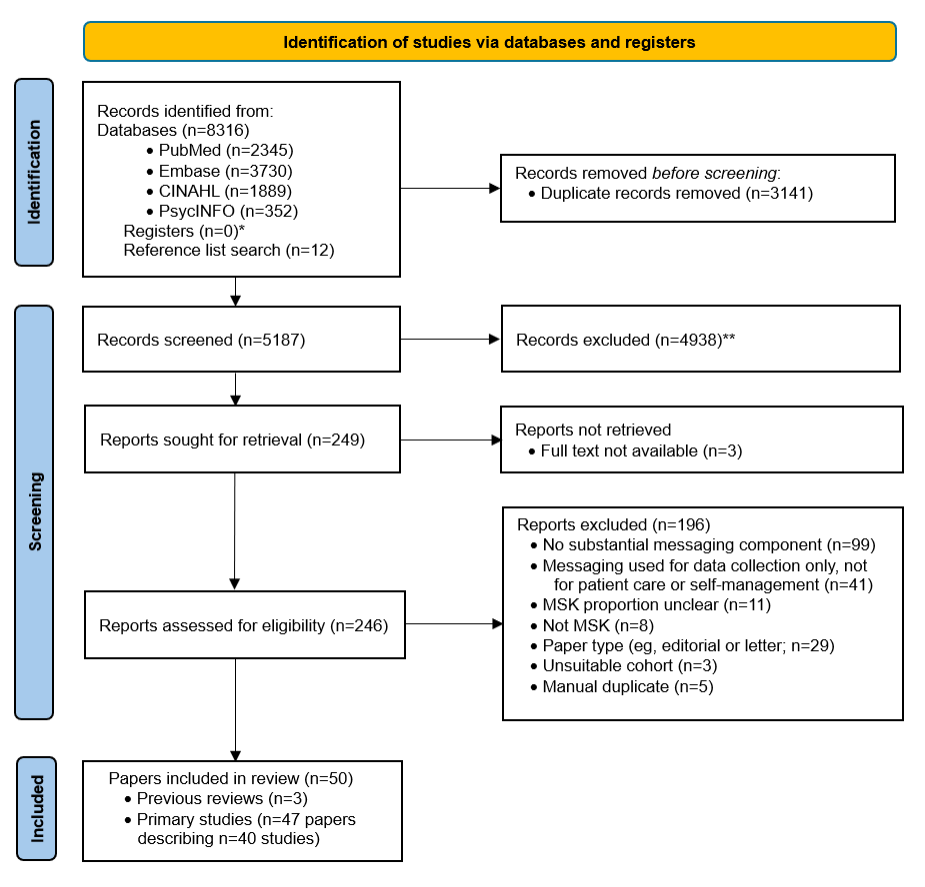
PRISMA (Preferred Reporting Items for Systematic Reviews and Meta-Analyses) flowchart—article selection process. *No registers were searched; **No automation tools were used; MSK: musculoskeletal.

Literature searches were conducted in August 2020 and repeated in May 2022. In this section, we present the combined results of both searches. We identified a total of 8328 papers (published in 2010-2022) for screening, of which 50 (0.6%) were included in this review. A PRISMA flowchart of the article selection process is shown in Figure 1.

We identified 3 previous systematic reviews, 2 (67%) of which we had already found while developing the protocol for this review [22,23] and 1 (33%) that was new [46]. One review focused specifically on the effects of text messaging for managing musculoskeletal pain conditions [22], while the remainder focused more broadly on digital health or mHealth for musculoskeletal conditions but covering some aspects of messaging [23,46]. The previous reviews were conducted in Australia, the United Kingdom, and the Netherlands, all countries classed as *very highly developed* according to their HDI. The characteristics of the reviews are shown in Table 1, and the findings are shown in Table 2. We did not identify any previous reviews related to design aspects of messaging for musculoskeletal pain conditions.

We included 47 papers describing 40 primary studies (22/40, 55% experimental; 16/40, 40% observational; and 2/40, 5% mixed methods). In total, 10% (4/40) of the experimental and observational studies had associated or embedded qualitative or mixed methods studies. The results of 5% (2/40) of the studies were multiply reported, and 8% (3/40) of the studies had either an associated design paper or a protocol paper containing design information. A total of 18 countries were represented, with the United States publishing the largest number of studies (9/40, 23%) followed by Australia (6/40, 15%) and Denmark (4/40, 10%). By HDI, most primary studies were conducted in *very highly developed* countries (36/40, 90%), 8% (3/40) were conducted in *highly developed* countries, and 3% (1/40) were conducted in a country of *medium development*. No studies were reported from countries of *low development*.

**Figure 2:**
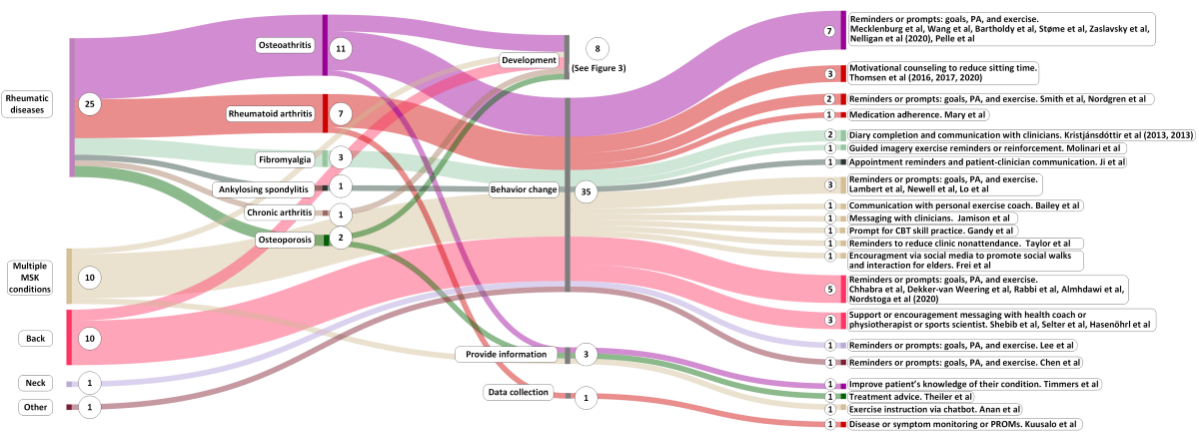
Overview of 47 papers describing 40 primary studies by condition, purpose, and role of messaging. The circled numbers represent the number of papers. CBT: cognitive behavioral therapy; MSK: musculoskeletal; PA: physical activity; PROM: patient-reported outcome measure.

At the time of our search, 70% (35/50) of the previous reviews and primary studies had been published in the 3 years before our search. The characteristics of the primary studies are shown in Table 3 and Figure 2 [24,26,27,30-32,35-38,47-83].

**Table 1:** Characteristics of review papers related to messaging for people with musculoskeletal (MSK) pain conditions.

Study, year; type	Country^a^ (HDI^b^)	Review focus	Studies and sample size	MSK condition focus	Primary outcomes	Messaging method		Adjunct
						SMS text messaging	Push notifications	
Fritsch et al [22], 2020; SR^c^	Australia (VH^d^)	Effects of text messaging for managing MSK pain	7 RCTs^e^; n=1181^f^	Any acute or chronic MSK^f^	Pain, function, adherence, and QoL^g^	✓	✓	Both
Hewitt et al [23], 2020; SR	United Kingdom (VH)	Digital health in the management of MSK conditions	19 RCTs; n=3361; 5 RCTs (n=1086) related to messaging	Any MSK condition excluding postsurgical management and pain related to computer use	Pain and functional disability; in addition, catastrophizing, self-efficacy, QoL, and coping strategies	✓	✓	Both
Seppen et al [46], 2020; ScR^h^	The Netherlands (VH)	Asynchronous mHealth^i^ interventions for RA^j^	10 studies; n=1214; 3 RCTs (n=266) related to messaging	RA	Medication compliance and sitting time	✓		Both

^a^On the basis of the lead author’s affiliation.

^b^HDI: Human Development Index [45].

^c^SR: systematic review.

^d^VH: very high.

^e^RCT: randomized controlled trial.

^f^Review included surgical studies; we report the subgroup of nonsurgical studies or participants in this table.

^g^QoL: quality of life.

^h^ScR: scoping review.

^i^mHealth: mobile health.

^j^RA: rheumatoid arthritis.

**Table 2:** Findings of review papers related to messaging for people with musculoskeletal (MSK) pain conditions.

Study, year	Individuals, problems, and purpose	Design-related information	Outcomes assessed and review findings
Fritsch et al [22], 2020—effects of text messaging for managing MSK pain	Review included 7 RCTs^a^ on patients with MSK pain conditions (3 with RA^b^, 1 with chronic widespread pain, 1 with upper- or lower-limb MSK injuries, 1 with frozen shoulder, and 1 with knee pain) [30-32,35-38,47] Messaging used to support behavior change. Most studies targeted physical activity or medication compliance.	Messaging features varied across studies. Examples include individualization to patient goals, timing, frequency, duration, directionality, and other intervention characteristics. The included studies provided little or limited description of the theoretical frameworks underpinning the interventions. Patient preferences were not described.	Clinical outcomes such as pain, function, disability, exercise adherence, QoL^c^, satisfaction with health care services, confidence in treatment, self-efficacy, and anthropometric measures Findings: Text messaging+UC^d^ vs UC No difference on pain [30] Equivocal or no difference on function [30,32] Equivocal or no difference on unscheduled appointments [31] Increase in calls to nurses [31] Messaging as part of the intervention vs any treatment: Pain: decrease [37,38]; equivocal or no difference [35,47] Function: equivocal or no difference [35,47]; increase [36,37] Exercise adherence: increase in self-reported adherence; equivocal or no difference on assessor-reported adherence [36] QoL: equivocal or no difference [31] SF-36^e^ MCS^f^: increase [35,37,47] SF-36 PCS^g^: increase [37]; equivocal or no difference [35,47] Comparison of messaging vs phone counseling Patient feedback and AEs^h^: assessed in 7 studies; AEs reported in 3 studies unrelated to messages
Hewitt et al [23], 2020—digital health for managing MSK conditions	Aspects of messaging were described in each of the following: 3 studies on self-management of back pain [24,25,27], 1 digitally delivered multidisciplinary pain program for back pain [28], and 1 conservative digital care program for knee pain [26].	Not described	Pain or function assessed via RCTs. Messaging (along with phone calls or email reminders) was described in the context of “additional efforts to encourage engagement” or “additional forms of support.” Review concluded that “additional forms of support” may be linked to positive outcomes (including improvement in pain and function); however, variability in messaging intervention characteristics hinders conclusions regarding effectiveness specific to messaging.
Seppen et al [46], 2020—asynchronous mHealth^i^ interventions for RA	Included 3 RCTs assessing the effectiveness of SMS text message reminders for medication adherence [32] and reducing sitting time [37,48].	Not described Some studies incorporated patients’ preferences; participants could select reminder frequency (1-5 per week) [37,48].	Messaging not evaluated directly; rather, patient outcomes relevant to the primary objective were assessed, such as medication compliance [32] and sedentary time [37,48]. Findings included the following: Increase in medication compliance^j^ [32] Reduced sitting time [37]

^a^RCT: randomized controlled trial.

^b^RA: rheumatoid arthritis.

^c^QoL: quality of life.

^d^UC: usual care.

^e^SF-36: 36-item Short-Form Health Survey.

^f^MCS: Mental Component Summary.

^g^PCS: Physical Component Summary.

^h^AE: adverse event.

^i^mHealth: mobile health.

^j^19-item Compliance Questionnaire on Rheumatology, incorrectly described as the 9-item Compliance Questionnaire on Rheumatology in the review by Seppen et al [46].

**Table 3:** Characteristics of primary studies related to messaging for people with musculoskeletal (MSK) pain conditions.

Study, year		Country^a^ (HDI^b^)	Design	Primary aim					Messaging method			Adjunct
				Provide information	Behavior change	Data collection	Design	SMS text messaging		Push notifications		
**Rheumatic diseases**												
	Kristjánsdóttir et al [35,47], 2013	Norway (VH^c^)	Experimental		✓			✓			Yes	
	Theiler et al [49], 2016	Switzerland (VH)	Observational	✓				✓			No	
	Thomsen et al [48], 2016	Denmark (VH)	Experimental		✓			✓			Yes	
	Mecklenburg et al [26], 2018	The United States (VH)	Experimental		✓			✓		✓	Yes	
	Molinari et al [50], 2018	Spain (VH)	Experimental		✓			✓			No	
	Nordgren et al [51], 2018, and Demmelmaier et al [75], 2015	Sweden (VH)	Observational, mixed methods study (stand-alone, associated, or embedded within a trial)		✓			✓			Yes	
	Timmers et al [52], 2018	The Netherlands (VH)	Experimental	✓						✓	Yes	
	Wang et al [38], 2018	Australia (VH)	Experimental		✓			✓			Yes	
	Bartholdy et al [53], 2019	Denmark (VH)	Experimental		✓			✓			No	
	Ravn Jakobsen et al [76], 2018	Denmark (VH)	Observational				✓^d^	✓			No	
	Geuens et al [77], 2019	Belgium (VH)	Mixed methods study (stand-alone, associated, or embedded within a trial)				✓^d^			✓	No	
	Ji et al [54], 2019	China (H^e^)	Observational		✓					✓	No	
	Mary et al [32], 2019	The United States (VH)	Experimental		✓			✓			Yes	
	Støme et al [55], 2019	Norway (VH)	Observational		✓					✓	Yes	
	Thomsen et al [37], 2017, and Thomsen et al [56], 2020	Denmark (VH)	Experimental		✓			✓			Yes	
	Zaslavsky et al [57], 2019	The United States (VH)	Observational		✓			✓			Yes	
	Kuusalo et al [31], 2020	Finland (VH)	Experimental			✓		✓			Yes	
	Nelligan et al [78], 2020 (qualitative study [stand-alone, associated, or embedded within a trial]), Nelligan et al [79], 2019 (qualitative study [stand-alone, associated, or embedded within a trial]), and Nelligan et al [58], 2019 (experimental)	Australia (VH)	Experimental and qualitative (stand-alone, associated, or embedded within a trial)		✓		✓^f^	✓			Yes	
	Pelle et al [59], 2020, and Pelle et al [80], 2019	The Netherlands (VH)	Experimental		✓		✓^f^			✓	No	
**Multiple MSK conditions**												
	Newell [60], 2012	Germany (VH)	Experimental		✓			✓			Yes	
	Taylor et al [61], 2012	Australia (VH)	Experimental		✓			✓			Yes	
	Gandy et al [62], 2016	Australia (VH)	Observational		✓			✓			Yes	
	Jamison et al [63], 2017	The United States (VH)	Experimental		✓					✓	Yes	
	Johnson et al [81], 2017	The United States (VH)	Observational				✓^d^	✓			Yes	
	Lambert et al [36], 2017	Australia (VH)	Experimental		✓					✓	Yes	
	Lo et al [64], 2018	China (H)	Observational		✓					✓	Yes	
	Frei et al [65], 2019	Switzerland (VH)	Mixed methods study (stand-alone, associated, or embedded within a trial)		✓					✓	Yes	
	Anan et al [66], 2021	Japan (VH)	Experimental	✓	✓					✓^g^	Yes	
	Bailey et al [67], 2020	The United States (VH)	Observational		✓			✓		✓	Yes	
**Low back pain**												
	Dekker-van Weering et al [68], 2015	The Netherlands (VH)	Observational		✓					✓	Yes	
	Chhabra et al [24], 2018	India (M^h^)	Experimental		✓					✓	Yes	
	Rabbi et al [69], 2018	The United States (VH)	Observational		✓					✓	No	
	Selter et al [70], 2018	The United States (VH)	Observational		✓					✓	No	
	Hasenöhrl et al [71], 2020	Austria (VH)	Observational and qualitative (stand-alone, associated, or embedded within a trial)		✓					✓	Yes	
	Shebib et al [27], 2019	The United States (VH)	Experimental		✓					✓	Yes	
	Almhdawi et al [72], 2020	Jordan (H)	Experimental		✓					✓	Yes	
	Nordstoga et al [73], 2020 (qualitative [stand-alone, associated, or embedded within a trial]), and Mork and Bach [82], 2018 (observational; protocol)	Norway (VH)	Observational and qualitative (stand-alone, associated, or embedded within a trial)		✓		✓^f^			✓	Yes	
	Fritsch et al [83], 2021	Australia (VH)	Observational				✓^f^	✓			No	
**Neck**												
	Lee et al [74], 2017	Korea (VH)	Experimental		✓			✓			Yes	
**Frozen shoulder**												
	Chen et al [30], 2017	Taiwan (VH)^d^	Experimental		✓			✓			Yes	

^a^On the basis of the lead author’s affiliation.

^b^HDI: Human Development Index [45].

^c^VH: very high.

^d^Mobile health design paper.

^e^H: high.

^f^Messaging-specific design paper.

^g^Messaging provided using a social media app.

^h^M: medium.

### RQ 1: Individuals, Problems, and Purpose

#### Previous Reviews

In the previous reviews [22,23,46] (Tables 1 and 2), the most commonly reported messaging interventions were for people with rheumatoid arthritis (RA) and back pain. For RA, messaging was used to monitor medication and disease activity [31] and improve medication adherence [32,46] and for reminders to reduce daily sitting time [37,46]. For people with back pain, messaging was used mostly as a component of self-management, with approaches focused on education and behavior change strategies [24,25], supportive messages provided by a health coach during periods of low engagement with a digital self-management program [27], and motivating messages sent as part of a multidisciplinary pain program [28].

Other studies described uses of messaging for people with knee pain, systemic lupus erythematosus, frozen shoulder, chronic widespread pain, and limb injuries or conditions. For knee pain, one study reported a lifestyle intervention focused on behavior change [22,38], and another reported participation reminders and app-based messaging with a personal coach as part of an exercise, education, or cognitive behavioral therapy (CBT) or weight loss or psychosocial support program [23,26]. For frozen shoulder, reminder, encouragement, and education messages were used to promote exercise compliance and improve shoulder function [30]. For chronic widespread pain, a CBT intervention used SMS text message diary completion prompts, with those diary entries then informing the treatment used by a therapist [35]. For limb injuries and conditions, messaging was used to promote adherence to a home exercise program in one study [36].

#### Primary Studies

Rheumatic diseases accounted for the largest proportion of the included primary studies (19/40, 48%), followed by studies on multiple musculoskeletal conditions or pain sites (10/40, 25%), back pain (9/40, 23%), neck pain (1/40, 3%), and “other” (1/40, 3%; Table 3).

##### Rheumatic Diseases

Of the 19 rheumatic disease–related studies, 8 (42%) focused on osteoarthritis [26,38,52,53,55,57-59,78-80], 5 (26%) focused on RA [31,32,37,48,51,56,75], 2 (11%) focused on fibromyalgia [35,47,50], 2 (11%) focused on osteoporosis [49,76], and 1 (5%) each focused on ankylosing spondylitis [54] and chronic arthritis [77].

Of these 19 studies, 14 (74%) described the use of messaging to promote behavior change with the intention of improving levels of physical activity, assisting weight loss, improving sleep, or reducing stress [26,32,35,37,38, 47,48,50,51,53-59,75,78-80]. A total of 11% (2/19) of the studies described messaging for providing information [49,52], and 5% (1/19) described the use of messaging to collect data for disease monitoring and guide clinical care [31]. In total, 26% (5/19) of the studies described aspects of design and development of messaging systems for people with knee osteoarthritis [79,80], osteoporosis [76], and chronic arthritis [77]. The design and development aspects are described in later sections.

##### Osteoarthritis Studies

Of the 8 studies on osteoarthritis, 2 (25%) focused on behavior change based on personalized goals. In the first study, which proposed personalized goals based on machine learning, participants were sent daily push notifications to remind them of their goals together with an interesting fact or answer to a frequently asked question [59,80]. Similarly, the second study used messaging to provide reminders to complete individualized physician-assigned goals and tasks, for which participants also used messaging to provide confirmation, or otherwise, that they had completed their personalized goals [55].

A total of 4 (50%) of studies focused on physical activity and exercise behavior change for people with knee osteoarthritis: of those, 1 (25%) used messages to decrease inactive behavior in people with knee osteoarthritis [53] and another (25%) used targeted personalized motivational reinforcement messages based on previous and current physical activity for people with osteoarthritis and sleep disturbance [57]. In the third study, which had an experimental design, the authors also explored patient attitudes and experiences of a self-directed digital health intervention incorporating automated messages to support strengthening exercises [78,79]. The fourth study, in which 77% of participants had knee osteoarthritis, described a digital care program that sent participants reminder messages if they did not engage with the program at the required intensity and also allowed participants to communicate with their health coach using messaging [26].

A single study focused on providing information for people with knee osteoarthritis, where messages were used to improve patients’ knowledge about their condition and treatment options before consultation with their specialist as part of shared decision-making [52].

A further study focused on knee osteoarthritis prevention, describing a self-management lifestyle intervention for young to middle-aged rural-dwelling women that incorporated messaging to provide key behavior reminders [38].

##### RA Studies

Of the 5 studies on RA, 2 (40%) used message reminders as part of a motivational counseling intervention to reduce sitting time [37,48,56], and 1 (20%) focused on physical activity behavior change with messaging used for coaching, prompts, reminders, and monitoring of physical activity program adherence [51,75]. A further study assessed the effects of text messages on medication adherence [32]. One study collected data using text or app-based messaging for symptom or disease monitoring and patient-reported outcome measures [31].

In a study that recruited women with chronic widespread pain (80% met the American College of Rheumatology criteria for fibromyalgia), text messaging was used to prompt diary completion and allow participants to exchange short messages with their therapist. The diary information was used by therapists to inform patient care [35,47]. A second guided imagery study also focused on people with fibromyalgia used text messaging to remind participants to practice their imaging exercises together with randomly selected reinforcement messages [50].

A study on patients with osteoporosis and nontraumatic fractures used text messaging to provide patients with treatment advice based on a validated fracture assessment tool and assessed whether the advice provided subsequently changed primary care physician management of their fracture [49].

Finally, one study described the use of social media messaging (WeChat) for people with ankylosing spondylitis, with messaging used for appointment reminders, for communication between physicians and patients, to record follow-up information, and for patients to provide feedback [54].

##### Multiple Musculoskeletal Conditions or Pain Sites

A total of 10 studies focused on multiple musculoskeletal conditions or pain sites (n=1, 10% each on the neck or back [64], neck, shoulder, or back [66], and chronic knee or low back pain [LBP] [67]). A total of 50% (5/10) of the studies recruited participants with a range of musculoskeletal problems typically seen in the general population [36,60,61,65], and 20% (2/10) of the studies recruited adults with chronic pain but not pain exclusively of musculoskeletal origin [62,63]. A further study focused on chronic musculoskeletal pain in veterans [81].

Of these 10 studies, 9 (90%) described behavior change interventions [36,60-63,65-67,81], and 1 (10%) was focused on providing information [66].

For neck and back pain, one study described the use of an artificial intelligence–enabled app that implemented evidence-based guidelines for self-management, with messaging provided within the app to remind participants to exercise and provide contact with the treating team [64]. A second study on workers with neck, shoulder, or back pain also described the use of artificial intelligence, wherein a chatbot provided messages with exercise instructions and suggestions for symptom improvement [66]. One study focused on chronic knee or LBP described a digital care program incorporating sensors and an app that allowed participants to communicate with a personal coach via SMS text messaging and app-based messaging [67].

Another 20% (2/10) of the studies included adults with chronic pain but not exclusively pain of musculoskeletal origin [62,63]. The first included patients being treated by a hospital-based pain management service for a range of conditions (LBP; cervical or upper-extremity, lower-extremity, abdominal or pelvic, and head or face pain; and multiple pain sites, with pain of ≥4 on a 0-10 scale). Participants used an app that incorporated reminders to complete daily assessments and also provided 2-way messaging [63]. The second study, with similar wide-ranging pain sites, used automated text messaging to prompt skill practice as part of an internet-delivered CBT program for chronic pain [62].

Regarding patients attending hospital physiotherapy services for a range of musculoskeletal problems, 10% (1/10) of the studies examined whether SMS text messaging could increase home exercise compliance [36]. In this study, compliance with exercises was encouraged via motivational SMS text messages sent by the physiotherapist. Similarly, the use of messaging to encourage home exercise compliance was described in a study on patients with musculoskeletal problems attending a chiropractic clinic [60].

In the physiotherapy outpatient setting, the use of SMS text message reminders to reduce clinic nonattendance was described in 10% (1/10) of the studies [61].

A total of 20% (2/10) of the studies focused on specific populations. The first, a community-based study, aimed to improve the physical activity of older adults (aged ≥60 years, most of whom had musculoskeletal problems) and used social media messaging (WhatsApp) to inform participants of scheduled walks and promote social interaction between participants [65]. The second study focused on a chronic musculoskeletal pain program in veterans and used behavior change messaging for stress management and adoption of healthy sleep practices and to increase engagement and retention in the program [81].

##### Back Pain

A total of 20% (8/40) of the studies described behavior change interventions [24,27,68-73,82], and 5% (2/40) described the design and development (described in a later section) [82,83]. Of the 8 behavior change studies, of these 4 (50%) described the use of individual or personalized messaging for physical activity goal reminders and reinforcement [24], encouragement messages and physical activity suggestions [69], motivational notifications for self-management [73,82], and individual activity level–based feedback messages provided on a PDA to encourage behavior change [68]. A total of 13% (1/8) of the studies described a self-management app with notifications to encourage walk breaks and posture exercises [72].

A total of 38% (3/8) of the studies described the use of 1- or 2-way messaging with a health coach, physiotherapist, or sports scientist for support, encouragement, and participation reminders as part of self-management programs [27,70,71].

##### Neck Pain

Only 3% (1/40) of the studies focused specifically on neck pain. This study described a behavior change intervention for office workers with chronic neck pain incorporating weekly messages about caring for their pain with information about the importance of exercise and to provide encouragement to complete prescribed exercises [74].

##### Other Conditions

A total of 3% (1/40) of the studies, on patients with frozen shoulder recruited from an orthopedic outpatient clinic, used messaging to provide reminders, encouragement, and education to promote shoulder exercise compliance [30].

### RQs 2 and 3: Design and Development and Patient Preferences

#### Overview

**Figure 3:**
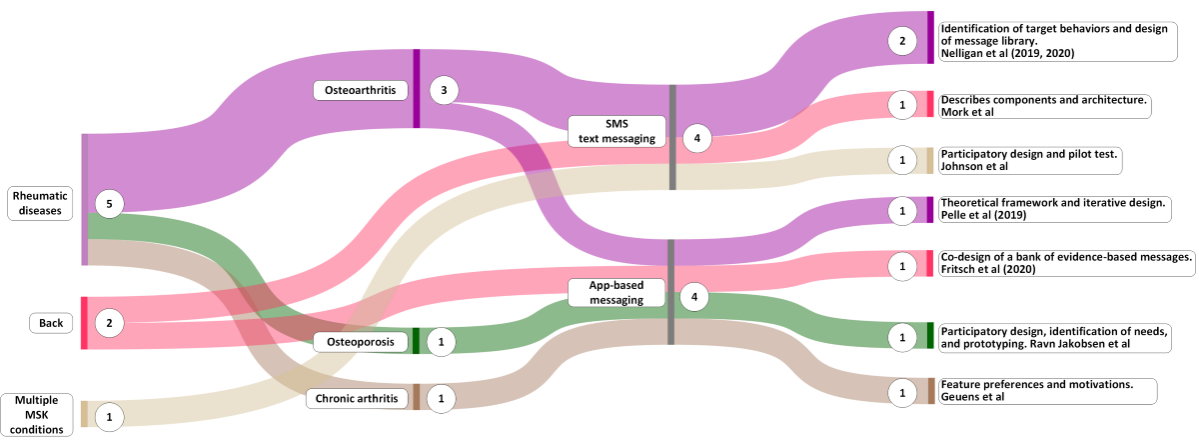
Overview of 8 papers describing aspects of design and development of messaging. The circled numbers represent the number of papers. MSK: musculoskeletal.

In this section, we report findings related to the design and development of messaging interventions. Because patient preferences, where accommodated, were generally addressed through participatory or co-design, we have reported the results of review questions 2 and 3 together. The findings are presented in three groups: (1) information found in papers specifically focused on the design and development of messaging interventions, (2) information found in mHealth design papers where some aspect of messaging was described alongside other mHealth functions, and (3) incidental design and development information found in papers that reported the results of messaging or mHealth interventions. The design-specific papers are shown in Table 4 and Figure 3 [76-83].

**Table 4:** Papers focused on messaging design and development and patient preferences.

Study, year	Role of messaging	Design process (theory, method, and outcomes)
Johnson et al [81], 2017—describes the participatory design and pilot study of an mHealth^a^ self-management program for veterans with chronic MSK^b^ pain	Tailored messages were an optional component intended to increase engagement and retention.	Participatory design involving a panel of veteran advisors, experts, and end users (number not specified). Input sought through interviews, focus groups, and usability testing but not described in detail. Messages were described as targeting behaviors, with message content and schedules matched to the participant’s stage of change based on the transtheoretical model of health behavior change [84]. The process through which the message content and schedules were derived was not described. Example messages included the following: “As a Veteran, you likely know many people who have or had pain. Think about one of them who could inspire you to manage your pain. Stress can make people more prone to pain. If you lower your stress, you can help lower your pain. See PAC activity Get the Facts [short-url].”
Mork and Bach [82], 2018 (protocol)—describes the components and architecture of an app-based self-management decision support system for LBP^c^ (selfBACK)	Messaging (via push notifications) used within the app to encourage physical activity	Authors stated that focus groups and iterative testing and development with patients, health professionals, and researchers were part of the development process without further detail. Structured intervention mapping [85], behavior change theories [86], and normalization process theory [87] During the development process, patients and health professionals (eg, physiotherapists and psychologists) were interviewed on their experience managing LBP. Educational content was reviewed by clinicians and researchers. Patient case data (baseline information, physical activity monitoring, and weekly patient-reported health and adherence outcomes) were used to generate motivational notifications to encourage physical activity. Little messaging-specific design information was provided.
Ravn Jakobsen et al [76], 2018—describes the participatory design and development of an mHealth app for women with newly diagnosed osteoporosis	Messaging used to communicate the results of a bone density scan (DXA^d^) to women and coordinate their follow-up appointment with their general practitioner.	A participatory design [88] was used. The team consisted of researchers, women, physicians, other health care professionals, and app designers. The iterative participatory app design process was somewhat unclear and described as commencing with 3 workshops (first, to generate ideas; second, to review wireframe designs; and third, to discuss the overall design content), followed by the creation of the design, feedback from users, development of a prototype, laboratory tests and feedback, adjustment, and final development. Messaging-specific design and development considerations were not described.
Nelligan et al [79], 2019—comprehensive description of the identification of behavior change targets; design of SMS text message library to support adherence to home exercise for people with knee OA^e^	SMS text message–based intervention Automated behavior change messages to promote exercise, with adaptive messages triggered by participant responses	Phase 1: theoretical rationale and application to inform the intervention SMS text messaging was selected as the mode of delivery based on literature describing it as a scalable, effective, efficient, and affordable way to promote adherence to health behaviors [19,89-94]. The authors used a previous scoping review [95] that mapped barriers and facilitators against the Theoretical Domains Framework [96]. Furthermore, the COM-B^f^ framework for understanding health behavior [97] and the BCW^g^ [97,98] were used throughout this phase. Previous work was used to identify messaging intervention functions appropriate for the SMS text messaging format [99]. Phase 2: development of SMS text messaging functions and a message library The SMS text messaging functionality was guided by the literature [100]. SMS text messages were automated and adaptive. Participants’ self-reported exercise adherence triggered a BCT^h^. The content of the messages was codeveloped by 7 academics, 4 physiotherapists, and 1 person with knee OA. The authors based their messaging frequency on previous literature, which, while inconsistent, suggests that 3 messages per week tapered over time was appropriate [92,100]. Examples are provided in appendixes accompanying the authors’ article [79] and in Table 5.
Geuens et al [77], 2019—identified feature preferences and motivations for a hypothetical self-management app for chronic arthritis	Messaging as a component in an app To provide medication or postural reminders	Authors referred to the PSD^i^ model [101]. Structured interviews to identify patient preferences for features in a hypothetical app and their motivations for selecting those features Limited messaging-specific information provided; however, reminders were rated highest in terms of desired features (medication and also posture). Praise and reward messages were considered less important, and social interaction features were rated the lowest.
Pelle et al [80], 2019—describes the theoretical framework and iterative design of an app for OA self-management	App was developed through an iterative design process that comprised medical researchers, physicians, physical therapists, patient representatives, and app developers.	Iterative development process involving researchers, health professionals, app designers, and patient representatives over 3-week “sprints” of development; user testing; reiteration; and, finally, pilot-testing After a review of the literature and consensus meetings, it was determined that motivation enhancement techniques such as reminders could increase the intervention effect. The Fogg Behavior Model [102], persuasive design [103], and daily push notifications to remind users of their goal and provide education on OA
Fritsch et al [83], 2021—described the co-design process for a bank of evidence-based messages for an LBP self-management text messaging intervention	Iterative codevelopment to identify relevant domains, content sources, frequency, appropriate timing, and a series of evidence-based messages for self-management of LBP	Behavior change methodology [104] previously used by Redfern et al [105,106] that links BCTs to frameworks such as information-motivation-behavior, theory of reasoned action, theory of planned behavior, social cognitive theory, control theory, and operant conditioning. 2-phase process previously used to develop messages in cardiology [106] conducted with consumers, researchers, and clinicians (n=39) to generate 82 messages Phase 1: development of concept and content with 15 consumers, clinicians, and researchers over 2 workshops to determine messaging features. In the workshop, it was decided that 4 weekly messages would be sent across the domains to provide education, motivation, or behavior change. Timing of appropriate messages for LBP self-management (9 AM, 12:30 PM, 4 PM, and 6 PM) was drawn from the literature [90,105]. Messages were subsequently drafted by 2 researchers and 2 consumer representatives and were then reviewed by 2 researchers with expertise in behavior change. Phase 2: iterative web-based review phase beginning with experts, then followed by consumers. Each message was reviewed by at least 2 participants in each round. Experts provided a score (mean 8.3/10) for appropriateness of content with consideration to current evidence and the likelihood of clinical effectiveness. Messages with a score of <8/10 (34%) were revised and then assessed by people with lived experience with LBP. These consumers scored each text messages on utility of content, understanding, and language acceptability. Messages with a score of <12/15 (31%) were revised according to feedback. Most frequently, consumer feedback focused on making the content more specific and less technical and including more examples.

^a^mHealth: mobile health.

^b^MSK: musculoskeletal.

^c^LBP: low back pain.

^d^DXA: dual-energy x-ray absorptiometry.

^e^OA: osteoarthritis.

^f^COM-B: Capability, Opportunity, and Motivation–Behavior.

^g^BCW: Behavior Change Wheel.

^h^BCT: behavior change technique.

^i^PSD: Persuasive System Design.

#### Papers Focused Specifically on Messaging Design and Development

A total of 4% (2/47) of the papers comprehensively described the design and development of SMS text messaging interventions for knee osteoarthritis [79] and back pain [83].

##### An SMS Text Messaging Intervention to Support Home Exercise Adherence for People With Knee Osteoarthritis

In 2019, Nelligan et al [79] comprehensively described a formal two-phase process to (1) identify behavior change targets and (2) design a library of SMS text messages to support adherence to home exercises for people with knee osteoarthritis. The development was guided by the recommended steps for developing text messaging–based programs for health behavior change published by Abroms et al [100] in 2015.

The first phase of development, comprising 3 stages, focused on target behavior, barriers, facilitators, and behavior change techniques using the Behavior Change Wheel framework [97,98]. Stage 1 drew on the literature to define the problem in behavioral terms, explaining the behavioral target and context and the barriers and facilitators for people with knee osteoarthritis in terms of participating in exercise mapped to domains in the Theoretical Domains Framework [96]. Barriers and facilitators relevant to the target behaviors were organized using the Capability, Opportunity, and Motivation–Behavior model for behavior change [97]. Stage 2 mapped barriers and facilitators to select intervention functions and behavior change techniques appropriate for implementation using SMS text messaging [93]. In stage 3, behavior change techniques for each function were identified from the Behavior Change Technique Taxonomy (version 1) [99].

The second phase involved the development of SMS text messaging functionality, specifically, a message library of content and determination of message frequency and level of interaction. Messaging content was derived by taking each barrier- or facilitator-linked behavior change technique identified in the first phase and constructing a relevant SMS text message. Message content was derived with input from 12 participants (1 person with knee osteoarthritis, 7 researchers, and 4 physiotherapists). In total, 3 authors derived the final message bank. A fourth author reviewed the final SMS text message wording to ensure that it was consistent with the Behavior Change Wheel mapping process and the identified behavior change techniques. The final message bank was organized into a 24-week schedule, assessed using literacy tools for readability, and tested by the authors for functionality and errors.

Author-provided examples of the mapping process and resulting SMS text message content for example barriers and facilitators are shown in Table 5.

**Table 5:** Example barrier and facilitator mapping process, abridged from Nelligan et al [79].

			COM-B^a^ category [97]		TDF^b^ domain [96]		Intervention function [99]		BCT^c^ [99]		Resulting SMS text message content
**Barrier mapping**											
	Forgetfulness	Psychological capability		10—memory, attention, and decision processes		Training		8.3—habit formation		“[Name], it can be hard to remember. We suggest making the exercises a habit. Set aside the same time each day to do them. It’s much harder to forget when something is a daily routine.”	
**Facilitator mapping**											
	Prioritizing exercise	Psychological capability		14—behavioral regulation		Enablement		10.9—self-reward		“Did you prioritize your exercises this week and get them done? Then reward yourself, [name]! Sticking to an exercise program for this long is a real accomplishment that deserves celebration.”	

^a^COM-B: Capability, Opportunity, and Motivation–Behavior.

^b^TDF: Theoretical Domains Framework.

^c^BCT: behavior change technique.

##### A Messaging Self-Management Intervention for LBP

In 2019, Fritsch et al [83] described the co-design process used to derive a bank of evidence-based lifestyle-focused messages for an LBP self-management text messaging intervention.

The authors used an iterative 2-phase co-design approach based on a framework used to design prevention messages for patients with cardiovascular disease previously published by Redfern et al [106] in 2014.

Phase 1 consisted of two 2-hour workshops intended to develop the concept, initial content, and messages. Workshop participants were researchers, clinicians with specific knowledge related to LBP, and consumer representatives from the support group Musculoskeletal Australia. At the first workshop, participants identified important domains relevant to LBP (exercise, education, mood, use of care, sleep, and medication) through reference to an evidence-based consumer resource (*Managing your pain: An A-Z guide*; Musculoskeletal Australia). The second workshop was focused on identifying sources of content for messages and duration, frequency, and timing of messages. Identified sources of content were relevant peer-reviewed literature, Australian and international clinical practice guidelines for LBP, and consumer group patient educational resources. Message frequency (4 messages per week) and timing (9 AM, 12:30 PM, 4 PM, and 6 PM) were based on previous work in coronary heart disease [107]. The development team considered that an intervention program duration of 12 weeks would be appropriate, with *exercise* domain messages being sent twice per week (emphasizing the importance of remaining active) and 1 message sent per week for each of the other domains.

This phase of the development process was also informed by previous work on factors related to engagement, perceived usefulness, behavior change, and delivery preferences for patients with coronary heart disease [105].

Following the workshops, a team comprising 2 researchers and 2 consumer representatives drafted evidence-based behavior change messages following the same theoretical approach by Redfern et al [106]. Messages were focused on education, motivation, or behavior change in the domains of *providing information or encouragement*; *prompting about consequences, intention formation, monitoring self-behavior, and barrier identification*; *advice about setting graded tasks*; and *strategies aimed at relapse prevention* and *the use of prompting and cues*. The team drafted an initial set of 82 positively phrased messages (by domain: 40 *exercise* messages, 10 *education* messages, 10 *mood* messages, 8 *use of care* messages, 7 *sleep* messages, and 7 *medication* messages) to take forward to the second phase of development.

In the second phase, the authors used a web-based survey of leaders in the field of LBP management to assess the appropriateness of the message content, gather opinions on the likelihood that the messages would be clinically effective, and make recommendations for message content improvement. The mean score for the messages from the expert review was 8.30/10. Messages with a score of <8/10 (34%) were modified in response to accompanying feedback. Subsequently, consumers scored each text message on utility of content, understanding, and language acceptability. Text messages with a consumer review score of <12/15 (31%) were revised according to feedback (mean score 12.5/15 points). Most frequently, consumer feedback focused on making the content more specific and less technical and including more examples.

#### Papers Describing the Design and Development of Messaging Within mHealth Apps

A total of 9% (4/47) of the papers described the design and development of more general mHealth interventions, where those interventions contained some use of messaging (alongside other mHealth features) for people with knee or hip osteoarthritis [80] and back pain [82], pain self-management for veterans [81], and women newly diagnosed with osteoporosis [76]. In total, 2% (1/47) of the papers focused on feature preferences for an app to support the self-management of chronic arthritis [77].

In each case, the design of the overall intervention was typically well described; however, the design of the content, timing, and frequency of the messaging components was not described in detail (Table 4). Because these papers provided little useful messaging-specific design and development information, we do not discuss them any further.

#### Incidental Design and Development Information Contained in Papers Reporting the Results of mHealth Interventions

We found little useful design-related information contained within the papers describing results of interventions. Typically, the papers described the purpose and behavior of the messaging component within their intervention, but the design processes used to determine message content, timing, and frequency were described incidentally or not at all (studies shown in Tables 6 and 7) [30,35,36,38,47,50,60,62,63,65,66,72,74]. For example, one paper provided examples of messages intended to provide encouragement, education, or motivation but provided no explanation of how these were derived [30]. Similarly, some papers (4/47, 9%) made a passing reference to co-design processes involving patients and clinicians but provided limited detail [37,48,55,56].

Some papers (17/47, 36%) described the use of messaging *adaptivity* (ie, dynamic system-initiated changes to the delivery of messaging to personalize content, frequency, or timing of messages based on automated or manual triggers) or *individualization*. Triggers for adaptivity included self-reported exercise adherence [79], automated physical activity data derived from wearables [57,68,73], self-reported data [54,66,71], personalized goals [37,48,55,56], and manual adaptivity triggers initiated by study personnel [31,63] and health coaches [26,27,67,70]. However, in these papers, no substantial detail was provided on the design considerations or processes related to the development of the intervention’s adaptive behavior.

**Table 6:** Messaging-specific intervention studies—efficacy and effectiveness.

Study, year	Objective	Duration and sample size (n)	Outcomes favoring messaging	Equivocal outcomes
Newell [60], 2012	Experimental study; for patients receiving chiropractic exercise advice, does text messaging with their practitioner, compared with no text messaging, improve exercise compliance?	4 weeks (32)	Self-reported exercise compliance (NRS^a^)	Patient-perceived practitioner care (NRS)
Taylor et al [61], 2012	Experimental study; for patients attending outpatient physical therapy clinics, do SMS text message reminders, compared with no reminders, reduce clinic nonattendance?	1 day (679)	Nonattendance at outpatient physiotherapy appointments (proportion)	Appointment attendance (proportion) Appointment cancellation (proportion)
Gandy et al [62], 2016	Observational study; for patients receiving an internet-delivered CBT^b^ program for pain, is the addition of message skill practice prompts, compared with no prompts, feasible and effective?	8 weeks (195)	Acceptability of SMS text messages (Likert scale)	Treatment satisfaction (Likert scale) Pain-related disability (RMDQ^c^) Depression (PHQ-9^d^) Anxiety symptoms (GAD-7^e^) Pain intensity (WBPQ^f^)
Theiler et al [49], 2016	Observational study; for patients with osteoporosis, do SMS text message reminders, compared with no reminders, improve adherence to drug therapy?	2 months (399)	Engagement with health care providers (proportion)	None
Chen et al [30], 2017	Experimental study; for patients with frozen shoulder, are reminder, encouragement, and educational messages delivered via mobile phone, compared with no messages, effective to increase exercise adherence and physical functioning?	2 weeks (66)	Patient-reported compliance with shoulder exercises^g^ Range of motion in forward flexion and internal and external rotation (goniometry) Patient satisfaction with SMS text messaging intervention (Likert scale)	Shoulder function (Simple Shoulder Test) Shoulder abduction (goniometry) Shoulder pain (VAS^h^)
Jamison et al [63], 2017	Experimental study; for patients with chronic pain using an app to record their progress, does 2-way supportive messaging, compared with no messaging, increase use or improve measures of pain or mood?	3 months (105)	Patient perceptions (more appealing, easier to use, easier to navigate, and less bothersome) Favored controls: participant perceptions of the responsiveness of providers to their reports	Frequency of use Pain (BPI^i^) Activity interference (PDI^j^) Mood (HADS^k^)
Timmers et al [52], 2018	Experimental study; for patients with knee osteoarthritis, does delivering education via an interactive mobile app, compared with standard education, increase patients’ knowledge of their illness and treatment options?	7 days (213)	Actual knowledge^l^ Perceived knowledge^l^ Patient satisfaction (NRS)	None
Bartholdy et al [53], 2019	Experimental study; for patients with knee osteoarthritis, do messages containing information and advice about the importance of performing daily activity, compared with no messages, lead to improved levels of activity?	6 weeks (38)	None	Time spent physically inactive, standing, and moving (accelerometry) Self-reported change in physical activity^m^ Pain severity, quality of life, and disability^n^
Mary et al [32], 2019	Experimental study; for patients with rheumatoid arthritis, compared with standard pharmacist consultation, does a 15-min pharmacist-led counseling session or message reminders improve methotrexate adherence?	6 months (96)	Medication adherence (CQR-19^o^) Patient satisfaction (Likert scale)	Medication adherence (GS^p^ and MPR^q^) Disease activityr
Kuusalo et al [31], 2020	Experimental study; for patients with rheumatoid arthritis, does using automated messages for enhanced monitoring, compared with routine care, improve disease activity and remission and quality of life?	6 months (166)	Physical functioning at 6 months after randomization (SF-36^r^) Health care resource use (nurse telephone contact)	Patients’ confidence in treatment (VAS) Physical and mental health–related quality of life (SF-36) Physical functioning at 12 months after randomization (SF-36) Disease activity^s^ Group rates of remission (proportion) Health care resource use^t^
Anan et al [66], 2021	Experimental study; for workers with neck and shoulder stiffness and pain or LBP^u^, does an AI^v^-assisted interactive health promotion system that operates through a mobile messaging app, compared with usual workplace exercise routine, lead to an improvement in musculoskeletal symptoms?	12 weeks (94)	Pain intensity (Likert scale) Perceived symptom improvement (Likert scale)	None

^a^NRS: numeric rating scale.

^b^CBT: cognitive behavioral therapy.

^c^RMDQ: Roland-Morris Disability Questionnaire.

^d^PHQ-9: 9-item Patient Health Questionnaire.

^e^GAD-7: 7-item Generalized Anxiety Disorder Scale.

^f^WBPQ: Wisconsin Brief Pain Questionnaire.

^g^Calculated as days answered “yes” to exercise/total days in the intervention.

^h^VAS: visual analog scale.

^i^BPI: Brief Pain Inventory.

^j^PDI: Pain Disability Inventory.

^k^HADS: Hospital Anxiety and Depression Scale.

^l^Customized scale (actual perceived level was measured on a 0-36 scale, or perceived level was measured on a 0-25 scale).

^m^Customized scale for change in self-reported physical activity (included no change, less time, or 0-3.5 more times compared to baseline).

^n^Knee Injury and Osteoarthritis Outcome Score.

^o^CQR-19: Compliance Questionnaire on Rheumatology.

^p^GS: Girerd score.

^q^MPR: medication possession ratio.

^r^SF-36: 36-item Short-Form Health Survey.

^s^Disease Activity Score–28 for Rheumatoid Arthritis, Health Assessment Questionnaire, erythrocyte sedimentation rate, and C-reactive protein.

^t^Except nurse telephone contact.

^u^LBP: low back pain.

^v^AI: artificial intelligence.

**Table 7:** Mobile health (mHealth) studies with an embedded messaging component.

Study, year	Duration and sample size (n)	Intervention and messaging features	Summary of findings
Kristjánsdóttir et al [35,47], 2013—short- and long-terms effects of a smartphone-based intervention with diaries and therapist feedback to reduce catastrophizing and increase functioning in women with chronic widespread pain	4 weeks (140)	Experimental study: Following a 4-week inpatient rehabilitation program, the study randomized participants to either a smartphone intervention or no smartphone intervention (controls). Follow-up occurred immediately after the intervention at 5 and 11 months. Smartphone intervention: Initial in-person session with a nurse to discuss functioning, health-related behavior goals, support needs, values, and value-based activities. Online web-based diaries completed 3 times a day on a smartphone covering pain interference, feelings and thoughts related to avoidance, catastrophizing and acceptance, planned and previous practice of self-management activities, and daily value-based and practical activities. Daily written situational feedback from a therapist based on the information entered in the diaries Audio files with guided mindfulness exercises SMS text messaging was used to prompt participants to complete their diaries and notify them when therapist feedback had been provided.	Reported between-group effects: Small effects on catastrophizing (PCS^a^) and value-based living (CPVI^b^) immediately after the intervention. Effect was nonsignificant at the 5-month follow-up. Moderate effect on acceptance (CPAQ^c^) immediately after the intervention and at the 5-month follow-up. Moderate effect on sleep disturbance (VAS^d^) and functioning and symptom severity [69] at the 5-month follow-up No effect on pain No significant between-group differences at the 11-month follow-up
Dekker-van Weering et al [68], 2015—pilot study of an activity-based feedback system for people with LBP^e^	15 days (17)	Observational study; participants’ daily activity was measured using a body-worn sensor. Real-time, hourly, personalized feedback was tailored to the individuals’ objectively measured activity level (eg, to discourage movement, to encourage movement, or a neutral message).	Encouraging feedback led to an increase in PA^f^. Discouraging feedback led to a decrease in PA. Greater participant response to feedback messages was associated with decreased pain scores.
Demmelmaier et al [75], 2015, and Nordgren et al [51], 2018—short- and longer-term (2-year) evaluation of an outsourced program to encourage PA in people with RA^g^	12 months (191)	Observational study Intervention: community-based exercise, support groups to facilitate behavior changes and feedback from physical therapists 2 messages each week were sent to collect data on how often the participant engaged in circuit training and moderately intense exercise.	Patients perceived the use of professional coaches and text messages to support the adoption of physical exercise as helpful. While improvements in self-reported physical activity, the proportion of participants who maintained increased physical activity, decreased significantly during year 2 of the study. Grip strength and quality of life reduced significantly during year 1 and 2 of the intervention. Reductions in activity limitation, systolic blood pressure and waist circumference were observed during second year. With most other health improvements sustained during year 1 and 2 of the study Participants reported that the text messages were a good reminder to engage in exercise (rated 4/5 on perceived value).
Thomsen et al [37], 2016; Thomsen et al [48], 2017; and Thomsen et al [56], 2020—evaluating the effect of motivational interviewing and messages on sitting time in patients with RA	4 months; follow-up: 10 and 22 months (150)	Experimental study; patients randomized to 3 individual motivational counseling sessions and messages aimed to reduce sedentary behavior (intervention) versus no contact and instructions to maintain usual lifestyle (controls). Manually created individual tailored messaging was used to remind participants of goals that they had set in their individual counseling sessions. Participants selected the frequency and timing of messages.	Reported between-group effects: Reduction in sitting time of –2.2 hours per day (95% CI –2.72 to –1.69) favoring the intervention Secondary measures, including fatigue, pain, self-efficacy, and HRQoL^h^, also favored the intervention.
Lambert et al [36], 2017—evaluating whether patients with MSK^i^ conditions have better adherence to home exercises when content is delivered via an app-based intervention compared to paper handouts	4 weeks (77)	Experimental study; participants randomized to receive home exercise program information via an app together with phone calls and motivational messages (intervention) versus paper handouts (controls) All participants were prescribed a 4-week exercise program.	Reported between-group effects: Small significant differences in adherence to the exercise program (NRS^j^; 1.3/11 points, 95% CI 0.2-2.3) and function (PSFS^k^; NRS 0.9/11 points, 95% CI 0.1-1.7) There were no significant differences in disability, patient satisfaction, perceived global impression of change, or assessor-reported adherence.
Lee et al [74], 2017—assessed the effectiveness of app-based exercises supported by weekly messages in office workers with chronic neck pain	8 weeks (20)	Small experimental pilot study in the workplace; participants randomized to receive prescribed exercises via a smartphone app (intervention) versus receiving a brochure and pain education (controls) Both groups received weekly education and encouragement messages.	Reported between-group effects: Statistically significant difference in pain intensity (VAS; 0-10) and functional disability (NDI^l^; expressed as a percentage); note: despite randomization, compared with controls, the intervention group had higher pain intensity at baseline (mean VAS score 5.20, SD 2.19 vs 4.02, SD 1.75) and a much higher NDI (mean 26.8, SD 9.68 vs 17.70, SD 9.20). No between-group differences in the secondary outcomes of strength, fear avoidance, and quality of life (SF-36^m^)
Chhabra et al [24], 2018—assessed the effect of a smartphone app on pain and function in patients with chronic LBP	12 weeks (93)	Experimental study; participants randomized to receive daily activity goals (back and aerobic exercise) in addition to written prescriptions (medication and recommended level of PA) provided through an app (intervention) versus written prescriptions only (controls) Activity goals were personalized based on participants’ health status, activities of daily living, and daily activity progress. Automated reinforcement messages were delivered via app push notifications.	Reported between-group effects: no significant difference in pain (NRS) and significant difference in disability (MODI^n^) favoring the intervention
Lo et al [64], 2018—assessed the feasibility of an AI^o^-embedded mHealth app for chronic neck and back pain in promoting self-management	Unclear (161)	Observational study Intervention: educational content including information on the pathophysiology of neck and back pain and principles of exercise for management of pain and coping strategies. Information was pushed via messages to participants’ social media accounts.	Pretest-posttest increase in time spent on rehabilitation exercises (custom questionnaire) Mean “self-reported improvement” of 65% (0-100 scale) Pretest-posttest reduction in pain from a median of 6 (IQR 5-8) to 4 (IQR 3-6; NRS 0-10) Perceived usability was 73/100 (cutoff for “acceptable” was 68/100; SUS^p^)
Mecklenburg et al [26], 2018—assessed the efficacy of a remotely delivered digital care program for chronic knee pain	12 weeks (162)	Experimental study; participants randomized to receive involved sensor-guided exercise therapy, psychoeducation, cognitive and behavioral therapy, and behavioral monitoring via the “Hinge Digital Care Program” (intervention) versus 3 digital education sessions and TAU^q^ (controls) The app included a coach and peer support discussion via messaging. Message or email reminders were sent if participants did not appear to engage at the recommended intensity of the program.	Reported between-group effects: Significant difference in pain and physical functioning (KOOS^r^), pain (VAS), and stiffness (VAS) favoring the intervention Interest in and the likelihood of needing surgery decreased, and patients’ understanding of their condition improved. Estimated surgery cost savings of US $4340 over 1 year and US $7900 over 5 years for participants who completed the digital care program compared to controls
Molinari et al [50], 2018—assessed the efficacy of using guided imagery to have patients with fibromyalgia picture their best possible selves	4 weeks (80)	Experimental study; participants randomized to receive “Best Possible Self,” a web-based app multimedia system to support patients through guided imagery (intervention) versus “Daily Activities” (active controls) The active control condition was not well described. Participants in both arms received 2 reminders each week via SMS text messaging prompting them to practice the guided imagery exercise.	Reported between-group effects: Postintervention improvements in depression, positive affect, and self-efficacy favoring “Best Possible Self” At the 30-month follow-up, there was improved optimism and negative affect favoring “Best Possible Self.”
Rabbi et al [69], 2018—evaluate the feasibility and acceptability of a personalized app for PA recommendations for adults with chronic pain	5 weeks (10)	Observational study Intervention: MyBehaviourCBP app, which generated PA recommendations based on sensor-detected PA. Recommendations were contextualized to the environment (road names), and new suggestions were continuations of the users’ repeated behaviors (eg, “Take walking break near Thompson St for 24minutes today”). Study comprised a 1-week period with no recommendations, 2 weeks with generic recommendations provided by an expert, and 2 weeks with automated recommendations.	Participants found the dynamic recommendations easier to adopt than the static generic recommendations. All participants found the recommendations “helpful.” Walking duration during the dynamic phase was greater than in the static phase (+4.9 min/d); no significant differences in pain (“Likert” scale; 0-10) and nonwalking exercise (min) were found. Qualitative feedback included that participants wanted notifications in the moment and adaptivity in relation to the weather or weekend days in addition to information related to the relationship between pain and activity levels.
Selter et al [70], 2018—described patient engagement and perceived utility and assessed the validity of a smartphone app module to quantify the functional status for people with chronic LBP	12 weeks (93)	Observational study Intervention: physical therapy program using the Limbr app involving 3 daily self-reports of pain and activity level and chat-based health coaching Health coaches monitored data and sent participants messages to provide support and remind them to interact with the program. Participants with low engagement (eg, only 1-2 interactive components per week) were sent weekly emails containing visual feedback on their use.	High level of attrition (38% completion rate); engagement was reported as “high amongst completers.” Depending on the type of self-report, 21%-32% interacted with the app. 76% of patients found that daily notifications helped them remember to complete their exercises, and 71% found that they helped them complete the daily surveys.
Wang et al [38], 2018—evaluated the effectiveness of a community-based self-management lifestyle program for young to middle-aged women with knee pain living in rural Australia	12 months (649)	Experimental study; participants randomized to receive 1 group session, monthly SMS text messages, 1 phone coaching session, and a program manual (intervention) versus 1 session of general women’s health education (controls) Program intended to improve lifestyle and prevent weight gain.	Reported between-group effects: Overall, no difference in the risk of knee pain worsening over 12 months For women who had knee pain at baseline (WOMAC^s^; 35% of participants), there was a lower risk of knee pain worsening over 12 months favoring the intervention, although this effect was only statistically significant for women with a BMI of ≥25 kg/m2 (OR^t^ 0.28, 95% CI 0.09-0.87).
Frei et al [65], 2019—assessed the effectiveness and feasibility and participant perceptions of a community-based PA intervention	12 months (29)	Observational study Intervention: the intervention facilitates and encourages participants to arrange walking groups within their local area. The messaging app, WhatsApp, was used to facilitate communication between the participants and study team.	Increased the minutes that participants engaged in moderate- to vigorous-intensity activity; no significant changes in step count 76% of participants reported that they attempted to recruit their peers to participate in the intervention. 62.4% of participants sent messages. Participants continued to organize walking groups via WhatsApp after the study team ceased their involvement.
Ji et al [54], 2019—described the design and preliminary evaluation of an interactive mHealth tool designed to help with the management and self-management of ankylosing spondylitis	13.3 months (1201)	Observational study Intervention: app designed to provide patient education on disease management and assist patients with medication adherence (SpAMS^u^) The tool consisted of a patient and physician portal and was linked to the social media app WeChat to allow for communication between physicians and patients, collect follow-up data, and obtain patient feedback.	Improvement in the proportion of patients with inactive disease or low disease activity from baseline to a mean follow-up time of 13.3 months (57.2%-79.2%) Problems solved using SpAMS avoided 29.1% of clinic visits. Average savings of 5.3 hours per patient in travel time and US $51 per person in personal expenses (15% of Chinese monthly disposable income) on physicians.
Shebib et al [27], 2019—evaluated the efficacy of a digital care program for patients with LBP	12 weeks (177)	Experimental study; participants randomized to receive a remotely administered digital care program that involved cognitive behavioral therapy, sensor-guided exercise therapy, education, symptom tracking, and unlimited personal coaching (intervention) versus 3 digital education articles and TAU (controls)	Reported between-group effects: improvements favoring the intervention in pain (MvK^v^: mean –16.4, 95% CI –22 to –10.9; VAS: mean –16, 95% CI –22.5 to –9.4), disability (MvK: mean –13, 95% CI –19.3 to –6.7; ODI^w^: mean –4.1, 95% CI –6.5 to –1.8), impact on daily life (VAS: mean –11/8, 95% CI –19.3 to –4.3), and understanding of their condition and treatment options (0-4; mean 0.5, 95% CI 0.2-0.7) and decreased interest in back surgery (mean –0.4, 95% CI –0.7 to –0.1)
Støme et al [55], 2019—feasibility study Investigating the acceptability, usability, and utility of a mobile app supporting goal achievement in patients with OA^x^	12 weeks (12)	Observational study Intervention: Vett app sent participants personal reminders to complete tasks that aligned with their PA, weight loss, and stress reduction goals. Participants were assigned 2 to 3 weekly physician-developed tasks and self-monitored their progress or received individualized feedback.	Primary reported outcome was mean goal achievement, which had a pretest-posttest improvement of 48%. Mean user satisfaction was 81/100, and technical usability was 80/100 to 84/100.
Zaslavsky et al [57], 2019—pilot study that assessed the feasibility and preliminary efficacy of a self-management mHealth intervention aimed at improving sleep among older adults with OA and disturbed sleep	19 weeks (24)	Observational study PA feedback based on wearable (Fitbit) data Participants received weekly personalized messages with motivational feedback in relation to their step count data. Participants who maintained or increased their step count received reinforcing messages. Those with declining step counts received encouraging messages. Participants also received motivational interviewing geared toward discussing the participants’ goals and strategies to facilitate behavior change.	Small pretest-posttest improvements in mean insomnia (ISI^y^; 1.2 points, 95% CI 2.45-0.05) and ASD^z^ (2.5 points, 95% CI 0.9-4.1) and self-reported overall sleep quality (derived from sleep diaries; 0.3 points, 95% CI 0.02-0.58) Nonsignificant improvements in step count, pain intensity, pain-related disability, self-efficacy, and sleep diary data and variables
Almhdawi et al [72], 2020—assessed the efficacy of an mHealth smartphone app in patients with LBP	6 weeks (39)	Experimental study; participants randomized to receive evidence-based instructions, therapeutic exercises, and reminders (intervention) versus instructions about nutrition (controls) Both arms received the app; the intervention group received reminders for walk breaks, posture, and exercises.	Reported between-group effects: Significant reductions (Cohen *d*) in pain intensity (VAS; 1.71, 0-11) and pain-related disability (ODI; 1.08) and improvements in physical quality of life (SF-12^aa^ PCS^ab^; 1.18) No significant differences in mental quality of life (SF-12 MCS^ac^); depression, anxiety, and stress symptoms (DASS-21^ad^); sleep quality (PSQI^ae^); and self-reported PA (IPAQ^af^)
Bailey et al [67], 2020—evaluated the efficacy of a digital care program in patients with chronic knee and back pain	12 weeks (10,264)	Observational study Intervention: Hinge Health app, which delivered education, sensor-guided exercise therapy (using a Bluetooth wearable sensor), behavioral health support, and 1:1 health coaching Patients were assigned a health coach, and communication occurred via SMS text messaging, email, or app-based messaging.	78% completed the program, with 69.6% achieving minimally important change in pain (20 points or 30% from baseline; VAS). Greater reduction in pain scores was associated with increasing levels of engagement in exercise therapy and participant-to-coach interactions. Significant reduction in the proportion of participants categorized as having depressive (PHQ-9≥5) or anxiety (GAD-7≥5) symptoms at 11 weeks compared with baseline (depression decreased by 57.9%, and anxiety decreased by 58.3%) Mean 1-year surgery likelihood score (subjective self-report response to the following question: “What do you think are the chances you’ll have [back/knee] surgery in the next year, in %?”; 0%-100%) decreased by 67.4% with respect to baseline.
Hasenöhrl et al [71], 2020—evaluated the feasibility and acceptance of orthopedists prescribing therapeutic exercises via a smartphone app to patients with nonspecific back pain	4 weeks (prestest-posttest assessment: 27 and semistructured interview: 16)	Small observational study with a qualitative component Intervention: individual physician-selected exercises sent via in-app messaging (n=27 participants). The physician could provide encouragement and mental support or unlock new exercises. Qualitative component: interviews and thematic analysis with a random sample of 16 of the 27 participants (research question not well described)	Reduction in mean hip circumference (–1.54, SD 2.75 cm) Reduction in back pain (ODI; mean –2.67, SD 4.99) Quality of life (SF-36): improved physical functioning (+5, SD 11.9); improved bodily pain (+14.8, SD 7.8); vitality (+7.2, SD 14.8) Participants reported that they would have preferred 2-way messaging
Nelligan et al [78], 2020 (qualitative), and Nelligan et al [58], 2021 (RCT^ag^)—explored the experiences and attitudes of patients with knee OA who participated in an mHealth intervention to support exercise	24 weeks (16)	Qualitative study (n=16 participants) embedded in an RCT with a targeted recruitment of n=206 Participants were randomized to receive website+SMS text messaging adherence support+home exercises (intervention) versus website only (controls). The website contained educational information (OA and exercise), PA recommendations, and prescription of knee-strengthening exercises. If participants adhered to the exercise program, they received a positive reinforcement message. If participants did not adhere, they were asked to select a barrier. All participants received behavior change techniques to assist with exercise adherence.	Five themes were reported: (1) technology was easy to use, (2) facilitators to exercise participation (credible information, website features, exercises that could be done unsupervised, and freedom to adapt exercises to suit needs), (3) sense of support and accountability (SMS text messaging served as a good reminder to engage in exercise, was easy to use, and held them accountable to weekly exercise; SMS text message tone and automation could trigger guilt or shame; and inability to contact someone when needed), (4) positive outcomes (symptom improvement, self-management confidence, and encouragement of active living), and (5) suggestions for real-world application (preference for provision by a health professional and should be subsidized or low cost). Primary outcomes favored in the intervention group in the RCT: decrease in pain scores (NRS; mean difference=1.6, 95% CI 0.9-2.22); increase in function (WOMAC; mean difference=5.2, 95% CI 1.9-8.5) Most secondary outcomes favored the intervention, which included KOOS pain, function in sport and recreation, ASES^ah^ pain and function subscales, AQOL-6D^ai^, and overall satisfaction (Likert scale). Changes in PASE^aj^, ASES function, and SEE^ak^ were similar between groups. Average participant message response rate was 73% (SD 7.5%), and 8% opted out. Patient perceptions (7-item Likert scale): mean perceived usefulness was 5.3 (SD 1.8), and mean agreement with message frequency was 5.3 (SD 1.7). Adverse events: 15.3% (intervention) vs 6.3% (control); a greater portion of the intervention group had knee pain (9.6%) compared to those in the control group (1.3%); a similar proportion used cointerventions throughout the study period.
Nordstoga et al [73], 2020, and Mork and Bach [82], 2018 (protocol)—evaluated the usability and acceptability of an mHealth intervention, selfBACK, in patients with LBP	4 weeks (16)	Observational study Intervention: the smartphone app provided participants with weekly self-management plans with content related to PA, flexibility exercises, and patient education. Behavior change techniques were incorporated into the app (eg, goal setting, feedback, monitoring, information about health consequences, and prompts). Motivational notification messages were sent to the participants’ smartphones.	Participants received an average of 1.8 notifications per day. Participants opened 42% of the notifications; of those opened, 90% were liked, and 8% were disliked; notifications of goal attainment were most frequently liked by participants. There was a lack of consensus on the frequency and appropriateness of motivational notifications. Motivational reminders served as facilitators of the intervention. 50% of the participants found the motivational messages useful. 30% of the participants found the notifications to be irrelevant and not functioning properly (eg, unsynchronized).
Pelle et al [59], 2020—investigated the effect of an mHealth intervention on secondary health care use in people with hip and knee OA	6 months (427)	Experimental study; participants assigned to receive a self-management app (Dr Bart mHealth app) intended to support goal setting and education and enhance motivation, with daily push notifications providing reminders on selected goals and educational information (intervention) versus TAU (controls) TAU consisted of any treatments initiated by participants.	Reported between-group effects: No difference in knee- or hip-related OA secondary health care use Significant group differences favoring the intervention were found between baseline and the 6-month follow-up for symptoms (mean difference=2.6, 95% CI 0.4-4.9), pain (mean difference=3.5, 95% CI 0.9-6.0), and activities of daily living (mean difference=2.9, 95% CI 0.2-5.6; HOOS^al^ and KOOS). No differences were found in any other outcome measures.

^a^PCS: Pain Catastrophizing Scale.

^b^CPVI: Chronic Pain Values Inventory.

^c^CPAQ: Chronic Pain Acceptance Questionnaire.

^d^VAS: visual analog scale.

^e^LBP: low back pain.

^f^PA: physical activity.

^g^RA: rheumatoid arthritis.

^h^HRQoL: health-related quality of life.

^i^MSK: musculoskeletal.

^j^NPRS: numeric rating scale.

^k^PSFS: Patient-Specific Functional Scale.

^l^NDI: Neck Disability Index.

^m^SF-36: 36-item Short-Form Health Survey.

^n^MODI: modified Oswestry Disability Index.

^o^AI: artificial intelligence.

^p^SUS: System Usability Scale.

^q^TAU: treatment as usual.

^r^KOOS: Knee Injury and Osteoarthritis Outcome Score.

^s^WOMAC: Western Ontario and McMaster Universities Osteoarthritis Index.

^t^OR: odds ratio.

^u^SpAMS: Smartphone Spondyloarthritis Management System.

^v^MvK: modified Von Korff scales.

^w^ODI: Oswestry Disability Index.

^x^OA: osteoarthritis.

^y^ISI: Insomnia Severity Index.

^z^ASD: acceptance of sleep difficulties.

^aa^SF-12: 12-item Short-Form Health Survey.

^ab^PCS: Physical Component Summary.

^ac^MCS: Mental Component Summary.

^ad^DASS-21: Depression, Anxiety, and Stress Scale–21.

^ae^PSQI: Pittsburgh Sleep Quality Index.

^af^IPAQ: International Physical Activity Questionnaire.

^ag^RCT: randomized controlled trial.

^ah^ASES: Arthritis Self-Efficacy Scale.

^ai^AQOL-6D: Assessment of Quality of Life.

^aj^PASE: Physical Activity Scale for the Elderly.

^ak^SEE: Self-Efficacy for Exercise.

^al^HOOS: Hip Injury and Osteoarthritis Outcome Score.

### RQs 4 and 5: Evaluation Methods, Efficacy, Effectiveness, and Economics

To avoid repetition, the findings of review questions 4 and 5 are reported together. A total of 28% (11/40) of the studies directly compared the use of messaging with an alternative; a further 60% (24/40) of the studies evaluated mHealth interventions with embedded use of messaging.

#### Studies Comparing the Use of Messaging With an Alternative

Of the 11 studies that directly compared messaging to an alternative, 9 (82%) had an experimental design and 2 (18%) were observational. In most cases, the comparator or control condition was no messaging or treatment as usual, with outcome measures varying by the intent of the intervention. Of these 11 studies, 3 (27%) [30-32] were described in the previous review on the effectiveness of text messaging interventions on the management of musculoskeletal pain [22], and the remainder were not, likely because they did not meet the inclusion criteria or were published later [49,52,53,60-63,66].

Overall, the outcomes either favored the messaging condition or were equivocal.

Examples of outcomes favoring messaging interventions included improved knowledge of the illness and the available treatment options and physical activity for knee osteoarthritis [52,53], improved medication adherence and physical functioning for RA [31,32], improved attendance to outpatient physiotherapy [61] and engagement with general practitioner [49], and improved exercise compliance for frozen shoulder [30] and mixed musculoskeletal conditions [60]. However, despite participants sometimes reporting messaging as *acceptable* [62] or *appealing* [63], and while improved pain intensity was found in participants with neck and shoulder pain and LBP [66], some studies (4/40, 10%) reported equivocal findings for important patient outcomes such as time spent physically active [53], pain [53,63], and quality of life [31,53].

In no studies did the primary outcome favor the control condition. In only one study, a secondary outcome (clinician responsiveness) favored the control condition. In this study, patients with chronic pain recorded their progress using an app, with intervention recipients also having access to messaging with their clinician (controls could report progress but had no messaging). Control participants perceived their clinicians to be more responsive to their progress reports [63].

No studies reported economic outcomes.

The studies are summarized in Table 6.

#### mHealth Studies With Embedded Messaging Components

A total of 24 studies evaluated mHealth interventions containing some form of embedded messaging component (n=11, 46% experimental; n=11, 46% observational; n=1, 4% observational with a qualitative component; and n=1, 4% qualitative embedded within an experimental study).

The results of efficacy and effectiveness were mixed, but because messaging was embedded within a larger mHealth intervention, it was not possible to isolate the messaging-specific effects from the overall intervention effects.

A total of 8% (2/24) of the studies reported economic outcomes—avoided surgery costs associated with a digital education program for chronic knee pain, in which messaging was used for coaching or peer support and program engagement reminders [26], and reduced travel time associated with a self-management mHealth tool for ankylosing spondylitis, in which social media messaging was used for communication between physicians and patients [54].

While it was not possible to isolate messaging-specific effects, these studies are included for completeness and summarized in Table 7.

## Discussion

### Principal Findings

To our knowledge, this is the first study to comprehensively map how mobile messaging has been used in the treatment and self-management of musculoskeletal conditions. We mapped the conditions and purposes for which messaging has been used and the approaches used to design and develop messaging interventions and summarized the evidence of efficacy, effectiveness, and economics from both experimental and observational studies. Our intent was to draw together all the available relevant information to help inform the future design of messaging interventions for people with musculoskeletal conditions and identify research gaps.

While previous reviews in this area are few, this work builds on 3 existing syntheses of the effectiveness of messaging interventions for people with musculoskeletal conditions. One review focused specifically on text messaging interventions for musculoskeletal pain [22]. The review included studies across a range of musculoskeletal problems and included both studies in which messaging was added to and compared with usual care (findings of positive effects only on exercise and medication adherence) and studies in which messaging was a component of a larger intervention (reporting some small effects on pain intensity, function, care-seeking behavior, exercise and medication adherence, and quality of life). Overall, the quality of the evidence was low. The 2 other reviews focused more generally on digital health for managing musculoskeletal conditions [23] and mHealth interventions for people with RA [46].

In this review, all the included studies that assessed intervention efficacy or effectiveness (on pain [30,53,63,66], function [30,31,63], disability [53], adherence to the intervention [30,60,63], physical activity levels [53], appointment attendance [49,61], health care contact [31], mood [63], quality of life [31,53], remission [31], and disease activity [31,32]) reported either equivocal findings or findings favoring messaging.

The notable absence of studies reporting negative outcomes may suggest publication bias. The lack of economic studies is also concerning; no messaging-specific studies reported economic outcomes. While, of the 40 studies, 2 (5%) digital or mHealth studies with messaging components did report economic outcomes, including avoided surgery costs and reduced travel time [26,54], the embedded nature of messaging means that it is not possible to attribute the observed savings specifically to the messaging components. While a previous review has shown messaging to be cost-effective in some circumstances, there is no information on the economic effects on musculoskeletal pain conditions; in cases in which messaging interventions are shown to be effective, further studies should be conducted to assess their economic effects [22,108].

We identified studies describing the use of messaging across a range of musculoskeletal conditions, with rheumatic diseases representing almost half (19/40, 48%) of the included studies, of which two-thirds (13/19, 68%) focused on osteoarthritis and RA. Somewhat surprisingly given its high population prevalence, back pain was represented by less than a quarter of primary studies (9/40, 23%). A further quarter of the studies (10/40, 25%) addressed multiple musculoskeletal conditions, but most (30/40, 75%) targeted single musculoskeletal conditions and pain sites despite evidence that musculoskeletal conditions often do not occur in isolation (eg, in Australia, 64% of people with back pain and 74% of people with arthritis have at least one other chronic condition) [3].

More than 80% of the included primary studies (34/40, 85%) focused on the use of prompts and reminder messages to foster positive behavior change at the individual level, most commonly in combination to encourage movement (eg, to increase physical activity, reduce sitting time, and improve compliance with prescribed exercise); compliance with prescribed medication; the practice of coping skills; and the meeting of personal goals. While a small number of studies (10/40, 25%) described the use of unidirectional or 2-way messaging with a health coach or exercise or sports scientist for support and encouragement, more studies (11/40, 28%) described the use of automated and unidirectional messaging, which, while economical on resources, may limit effectiveness in fostering behavior change.

While most studies (34/40, 85%) focused on influencing individual behavior change, there appears to be limited research into the use of messaging to improve treatment or self-management at the broader system level (eg, to improve health care processes, handover communication, and continuity of care between providers). One study on RA used SMS text messaging–based monitoring of medication adherence and disease progression to inform follow-up nurse contact but found no difference in the primary outcome of remission [31]. A second study on a digital health platform for ankylosing spondylitis management consisting of a patient and physician portal and 2-way chat via social media reported improvements in the proportion of patients with inactive disease and an avoidance of 29.1% of in-person clinic visits [54]. Future studies could focus on addressing the gaps in knowledge on more process- or system-focused interventions.

Overall, we found limited information about messaging design. One study (1/40, 3%) used development processes previously described by Redfern et al [106], which, while originally focused on cardiovascular event prevention messaging, have since been more widely adopted and adapted in the co-design of text messaging interventions, including for diabetes prevention [109], endometriosis support [110], and support after breast cancer treatment [111]. Similarly, one study (1/40, 3%) used guidance for the development and testing of messaging in health behavior change developed by Abroms et al [100].

Only 4% (2/47) of the papers provided comprehensive information about message design and development [79,83] and highlighted the importance of taking a formal approach and having a theoretical underpinning and meaningful consumer involvement. Beyond these 2 papers, design was typically poorly described or not described at all, and many projects appeared to leapfrog from concept to implementation with a limited or absent design phase, perhaps not recognizing the importance of formal design for subsequent adoption.

While some papers (8/47, 17%) did describe elements of participatory design or co-design, some papers (4/47, 9%) provided limited detail or had limited consumer involvement, or in some cases, consumers were involved after the design had already been conceived by researchers and clinicians. In most cases, we found that papers described the theoretical basis underlying their intended behavior change well, but consistent with a previous review [22], we found few detailed descriptions of the process through which the content, timing, and frequency of the messages were derived. This remains an important weakness in the musculoskeletal literature specifically and has been identified more generally by others [100,106]. Further work should be conducted to elicit preferences regarding these processes from people across the spectrum of musculoskeletal conditions.

The strengths of this review include our comprehensive search strategy and the inclusion of a wide range of studies and designs, providing a rich map of the literature expanding the insights provided by previous effectiveness-focused reviews. A limitation is that we focused specifically on the use of messaging in patient care and self-management, and it is possible that there are other messaging applications relevant to people with musculoskeletal conditions. However, we made this review as broad as possible within available resources. Because a comprehensive synthesis was time-consuming and we last conducted the searches in 2022, there may be important, more recent studies that we missed in this review. Similarly, resources did not allow us to review the gray literature.

### Conclusions

In conclusion, messaging has been used for the care and self-management of a range of musculoskeletal conditions with generally favorable outcomes reported. Nonetheless, there are areas that should be addressed by future research to improve the quality of intervention design, which will hopefully translate into uptake and sustainability. First, preferences related to messaging content, timing, and frequency should be further explored specifically among people with musculoskeletal conditions, eliminating the reliance on information from other disciplines. Second, teams should incorporate digital intervention design expertise, follow formal design processes, and clearly describe design considerations and processes used. Finally, in cases in which messaging interventions are shown to be effective, further studies should be conducted to assess their economic effects and practical considerations related to implementation and sustainability.

## Appendix

Multimedia Appendix 1 Search strategy.

Multimedia Appendix 2 PRISMA-ScR Checklist.

## Abbreviations

CBT: cognitive behavioral therapy
HDI: Human Development Index
LBP: low back pain
mHealth: mobile health
PRISMA: Preferred Reporting Items for Systematic Reviews and Meta-Analyses
PRISMA-ScR: Preferred Reporting Items for Systematic Reviews and Meta-Analyses extension for Scoping Reviews
RA: rheumatoid arthritis
RCT: randomized controlled trial
RQ: research question
