# Supplementary material for: Development and Use of Mobile Messaging for Individuals With Musculoskeletal Pain Conditions: Scoping Review
Source: JMIR Mhealth Uhealth. 2024 Aug 14;12:e55625. doi: 10.2196/55625 (PMC11358670; doi:10.2196/55625)
Supplement: Multimedia Appendix 1 [file mhealth_v12i1e55625_app1.docx]

**Supplementary Materials – search strategy**

Search years presented are as per originally published protocol, an updated search was conducted in 2022 as described in the main manuscript.

Table S1. Search strategy for Medline via PubMed

| **Search** | **Query** |
| --- | --- |
| #1 | Back [tiab] OR neck [tiab] OR thoracic [tiab] OR lumbar [tiab] OR knee [tiab] OR shoulder [tiab] OR elbow [tiab] OR hand [tiab] OR wrist [tiab] OR foot [tiab] OR ankle [tiab] OR hip [tiab] OR musculoskeletal [tiab] OR muscle [tiab] OR ligament [tiab] OR tendon [tiab] OR "soft tissue" [tiab] |
| #2 | pain [tiab] OR injury [tiab] OR injuries [tiab] OR sprain* [tiab] OR strain* [tiab] OR tear [tiab] |
| #3 | #1 AND #2 |
| #4 | Backache [tiab] OR musculoskeletal [tiab] OR neckache [tiab] OR whiplash [tiab] OR arthri* [tiab] OR osteoarthritis [tiab] |
| #5 | Back Pain [mh] OR Neck Pain [mh] OR Shoulder Pain [mh] OR Musculoskeletal Pain [mh] OR Musculoskeletal Diseases [mh] OR Back Injuries [mh] OR Neck Injuries [mh] OR Thoracic Injuries [mh] OR Leg Injuries[mh] OR Shoulder Injuries [mh] OR Hand Injuries [mh] OR Hip Injuries [mh] OR Tendon Injuries [mh] OR Soft Tissue Injuries [mh] OR Sprains and Strains [mh] OR Arm Injuries [mh] OR Accidents [mh] |
| #6 | #3 OR #4 OR #5 |
| #7 | "Text message" [tiab] OR "text messages" [tiab] OR "text reminder" [tiab] OR "text reminders" [tiab] OR texting [tiab] OR SMS [tiab] OR "short message service*" [tiab] OR messaging [tiab] OR "mobile phon*" [tiab] OR smartphon* [tiab] OR "cellular phon*" [tiab] OR "cell phone" [tiab] OR "smart phone" [tiab] OR mhealth [tiab] |
| #8 | cell phone [mh] or mobile applications [mh] |
| #9 | #7 OR #8 |
| #10 | #6 AND #9 |
| Limited to: In the last 10 years, English. | |

Table S2. Search strategy for CINAHL via EBSCOhost

| **Search** | **Query** |
| --- | --- |
| S1 | TI Back OR neck OR thoracic OR lumbar OR knee OR shoulder OR elbow OR hand OR wrist OR foot OR ankle OR hip OR musculoskeletal OR muscle OR ligament OR tendon OR "soft tissue" |
| S2 | AB Back OR neck OR thoracic OR lumbar OR knee OR shoulder OR elbow OR hand OR wrist OR foot OR ankle OR hip OR musculoskeletal OR muscle OR ligament OR tendon OR "soft tissue" |
| S3 | S1 OR S2 |
| S4 | TI pain OR injury OR injuries OR sprain* OR strain* OR tear |
| S5 | AB pain OR injury OR injuries OR sprain* OR strain* OR tear |
| S6 | S4 OR S5 |
| S7 | S3 AND S6 |
| S8 | TI Backache OR musculoskeletal OR neckache OR whiplash OR arthri* OR osteoarthritis |
| S9 | AB Backache OR musculoskeletal OR neckache OR whiplash OR arthri* OR osteoarthritis |
| S10 | S8 OR S9 |
| S11 | (MH "Back Pain+") OR (MH "Neck Pain") OR (MH "Shoulder Pain") OR (MH "Elbow Pain") OR (MH "Muscle Pain") OR (MH "Knee Pain+") OR (MH "Musculoskeletal Diseases+") OR (MH "Back Injuries+") OR (MH "Neck Injuries+") OR (MH "Ligament Injuries+") OR (MH "Soft Tissue Injuries+") OR (MH "Thoracic Injuries+") OR (MH "Leg Injuries+") OR (MH "Hand Injuries+") OR (MH "Tendon Injuries+") OR (MH "Soft Tissue Injuries+") OR (MH "Sprains and Strains+") OR (MH "Arm Injuries+") OR (MH "Accidents+") |
| S12 | S7 OR S10 OR S11 |
| S13 | TI "Text message" OR "text messages" OR "text reminder" OR "text reminders" OR texting OR SMS OR "short message service*" OR messaging OR "mobile phon*" OR smartphon* OR "cellular phon*" OR "cell phone" OR "smart phone" OR mhealth |
| S14 | AB "Text message" OR "text messages" OR "text reminder" OR "text reminders" OR texting OR SMS OR "short message service*" OR messaging OR "mobile phon*" OR smartphon* OR "cellular phon*" OR cell phone OR "smart phone" OR mhealth |
| S15 | S13 OR S14 |
| S16 | (MH "Cellular Phone+") OR (MH "Mobile Applications") |
| S17 | S15 OR S16 |
| S18 | S12 AND S17 |
| Limited to: Published Date: 20100101-20201231; English Language. | |

Table S3. Search strategy for EMBASE

| **Search** | **Query** |
| --- | --- |
| #1 | Back:ab,ti,kw OR neck:ab,ti,kw OR thoracic:ab,ti,kw OR lumbar:ab,ti,kw OR  knee:ab,ti,kw OR shoulder:ab,ti,kw OR elbow:ab,ti,kw OR hand:ab,ti,kw OR  wrist:ab,ti,kw OR foot:ab,ti,kw OR ankle:ab,ti,kw OR hip:ab,ti,kw OR  musculoskeletal:ab,ti,kw OR muscle:ab,ti,kw OR ligament:ab,ti,kw OR tendon:ab,ti,kw  OR 'soft tissue':ab,ti,kw |
| #2 | pain:ab,ti,kw OR injury:ab,ti,kw OR injuries:ab,ti,kw OR sprain*:ab,ti,kw OR  strain*:ab,ti,kw OR tear:ab,ti,kw |
| #3 | #1 AND #2 |
| #4 | Backache:ab,ti,kw OR musculoskeletal:ab,ti,kw OR neckache:ab,ti,kw OR  whiplash:ab,ti,kw OR arthri*:ab,ti,kw OR osteoarthritis:ab,ti,kw |
| #5 | 'musculoskeletal disease'/exp OR 'accidental injury'/exp OR 'head and neck injury'/exp  OR 'soft tissue injury'/exp OR 'leg injury'/exp OR 'arm injury'/exp |
| #6 | #3 OR #4 OR #5 |
| #7 | 'Text message':ab,ti,kw OR 'text messages':ab,ti,kw OR 'text reminder':ab,ti,kw OR 'text reminders':ab,ti,kw OR texting:ab,ti,kw OR SMS:ab,ti,kw OR 'short message service*':ab,ti,kw OR messaging:ab,ti,kw OR 'mobile phon*':ab,ti,kw OR smartphon*:ab,ti,kw OR 'cellular phon*':ab,ti,kw OR 'cell phone':ab,ti,kw OR 'smart phone':ab,ti,kw OR mhealth:ab,ti,kw |
| #8 | 'text messaging'/exp OR 'mobile phone'/exp OR 'mobile application'/exp |
| #9 | #7 OR #8 |
| #10 | #6 AND #9 |
| Limited to: 2010-2020, English, Embase. | |

Table S4. Search strategy for PsycINFO via APA PsycNet

| **Search** | **Query** |
| --- | --- |
| #1 | Title: Back OR Title: neck OR Title: thoracic OR Title: lumbar OR Title: knee OR Title: shoulder OR Title: elbow OR Title: hand OR Title: wrist OR Title: foot OR Title: ankle OR Title: hip OR Title: musculoskeletal OR Title: muscle OR Title: ligament OR Title: tendon OR Title: "soft tissue" |
| #2 | Abstract: Back OR Abstract: neck OR Abstract: thoracic OR Abstract: lumbar OR Abstract: knee OR Abstract: shoulder OR Abstract: elbow OR Abstract: hand OR Abstract: wrist OR Abstract: foot OR Abstract: ankle OR Abstract: hip OR Abstract: musculoskeletal OR Abstract: muscle OR Abstract: ligament OR Abstract: tendon OR Abstract: "soft tissue" |
| #3 | #1 OR #2 |
| #4 | Title: pain OR Title: injury OR Title: injuries OR Title: sprain* OR Title: strain* OR Title: tear |
| #5 | Abstract: pain OR Abstract: injury OR Abstract: injuries OR Abstract: sprain* OR Abstract: strain* OR Abstract: tear |
| #6 | #4 OR #5 |
| #7 | #3 AND #6 |
| #8 | Title: Backache OR Title: musculoskeletal OR Title: neckache OR Title: whiplash OR Title: arthri* OR Title: osteoarthritis |
| #9 | Abstract: Backache OR Abstract: musculoskeletal OR Abstract: neckache OR Abstract: whiplash OR Abstract: arthri* OR Abstract: osteoarthritis |
| #10 | #8 OR #9 |
| #11 | Index Terms: Back Pain OR Index Terms: whiplash OR Index Terms: Injuries OR Index Terms: Musculoskeletal Disorders OR Index Terms: Accidents |
| #12 | #7 OR #10 OR #11 |
| #13 | Title: "Text message" OR Title: "text messages" OR Title: "text reminder" OR Title: "text reminders" OR Title: texting OR Title: SMS OR Title: "short message service*" OR Title: messaging OR Title: "mobile phon*" OR Title: smartphon* OR Title: "cellular phon*" OR Title: "cell phone" OR Title: "smart phone" OR Title: mhealth |
| #14 | Abstract: "Text message" OR Abstract: "text messages" OR Abstract: "text reminder" OR Abstract: "text reminders" OR Abstract: texting OR Abstract: SMS OR Abstract: "short message service*" OR Abstract: messaging OR Abstract: "mobile phon*" OR Abstract: smartphon* OR Abstract: "cellular phon*" OR Abstract: "cell phone" OR Abstract: "smart phone" OR Abstract: mhealth |
| #15 | #13 OR #14 |
| #16 | Index Terms: Cell phones OR Index Terms: Mobile Applications OR Index Terms: Text Messaging OR Index Terms: Mobile Assessment |
| #17 | #15 OR #16 |
| #18 | #12 AND #17 |
| #19 | Year: 2010 to 2020 |
| Limited to: Language English. | |
